# Integrative analysis of DNA methylation in discordant twins unveils distinct architectures of systemic sclerosis subsets

**DOI:** 10.1186/s13148-019-0652-y

**Published:** 2019-04-04

**Authors:** Paula S. Ramos, Kip D. Zimmerman, Sandra Haddad, Carl D. Langefeld, Thomas A. Medsger, Carol A. Feghali-Bostwick

**Affiliations:** 1 Division of Rheumatology and Immunology, Department of Medicine, Medical University of South Carolina, Charleston, SC USA; 2 Department of Public Health Sciences, Medical University of South Carolina, Charleston, SC USA; 3 Department of Biostatistics and Data Science, Wake Forest School of Medicine, Winston-Salem, NC USA; 4 Center for Public Health Genomics, Wake Forest School of Medicine, Winston-Salem, NC USA; 5 Bay Path University, Longmeadow, MA USA; 6 Division of Rheumatology and Clinical Immunology, Department of Medicine, University of Pittsburgh, Pittsburgh, PA USA

**Keywords:** Systemic sclerosis, DNA methylation, Genome, Blood, Twins

## Abstract

**Background:**

Systemic sclerosis (SSc) is a rare autoimmune fibrosing disease with an incompletely understood genetic and non-genetic etiology. Defining its etiology is important to allow the development of effective predictive, preventative, and therapeutic strategies. We conducted this epigenomic study to investigate the contributions of DNA methylation to the etiology of SSc while minimizing confounding due to genetic heterogeneity.

**Methods:**

Genomic methylation in whole blood from 27 twin pairs discordant for SSc was assayed over 450 K CpG sites. In silico integration with reported differentially methylated cytosines, differentially expressed genes, and regulatory annotation was conducted to validate and interpret the results.

**Results:**

A total of 153 unique cytosines in limited cutaneous SSc (lcSSc) and 266 distinct sites in diffuse cutaneous SSc (dcSSc) showed suggestive differential methylation levels in affected twins. Integration with available data revealed 76 CpGs that were also differentially methylated in blood cells from lupus patients, suggesting their role as potential epigenetic blood biomarkers of autoimmunity. It also revealed 27 genes with concomitant differential expression in blood from SSc patients, including *IFI44L* and *RSAD2*. Regulatory annotation revealed that dcSSc-associated CpGs (but not lcSSc) are enriched at Encyclopedia of DNA Elements-, Roadmap-, and BLUEPRINT-derived regulatory regions, supporting their potential role in disease presentation. Notably, the predominant enrichment of regulatory regions in monocytes and macrophages is consistent with the role of these cells in fibrosis, suggesting that the observed cellular dysregulation might be, at least partly, due to altered epigenetic mechanisms of these cells in dcSSc.

**Conclusions:**

These data implicate epigenetic changes in the pathogenesis of SSc and suggest functional mechanisms in SSc etiology.

**Electronic supplementary material:**

The online version of this article (10.1186/s13148-019-0652-y) contains supplementary material, which is available to authorized users.

## Background

Systemic sclerosis (SSc or scleroderma) is a rare multisystem, connective tissue disease characterized by cutaneous and visceral fibrosis, immune dysregulation, and vasculopathy. Patients are commonly classified into two main clinical subsets on the basis of the extent of skin thickening: limited or restricted cutaneous SSc (lcSSc) and diffuse or widespread cutaneous SSc (dcSSc). The etiology of SSc remains elusive. The low concordance rate in monozygotic twins and relatively modest genetic burden suggest a substantial role for epigenetic or environmental factors in SSc susceptibility [1, 2]. Environmental factors (e.g., nutrition, behavior, stress) can influence methylation and other epigenetic marks that result in phenotypic change and disease [3]. Thus, epigenetic variation may play an important role in SSc risk.

DNA methylation is a chemical modification of cytosine bases generally associated with transcriptional repression when at regulatory elements such as promoters and enhancers [4, 5]. Nevertheless, the precise relationships between DNA methylation and gene expression are complex and poorly understood [5–8]. The correlation between DNA methylation and gene expression can be positive or negative and is tissue-specific and context-specific, in that the local DNA sequence and genomic features largely account for local patterns of methylation [4, 9–11]. In addition to its potential to affect an individual’s susceptibility to SSc, changes in the methylation of DNA may occur secondarily to SSc and may consequently influence disease progression. There is compelling evidence that DNA methylation plays a role in the pathogenesis of autoimmune diseases, and multiple epigenome-wide association studies revealed the existence of differentially methylated regions associated with, for example, systemic lupus erythematosus (SLE) [12–17], rheumatoid arthritis [18–26], or psoriasis [27–32]. In SSc, differentially methylated genes were reported in an X chromosome analysis of peripheral blood mononuclear cells [33] and in one genome-wide DNA methylation analysis in dermal fibroblasts [34]. Disease-discordant monozygotic twins offer the ideal study design to investigate the association of DNA methylation with a disease, as it minimizes confounding due to genetic heterogeneity, sex-, age- and early-life environmental effects [35, 36].

To our knowledge, no genome-wide investigation of DNA methylation in whole blood from discordant twins has been reported in SSc. We first conducted epigenomic profiling to investigate the association between DNA methylation variation and SSc. Next, we conducted tissue-specific regulatory annotation and integration with available data from DNA methylation and gene expression profiling studies, with the goal of gaining insights into the potential molecular mechanisms underlying SSc development and/or progression.

## Methods

### Subjects

A total of 27 twin pairs discordant for SSc were used for this study (Table 1). All subjects have been previously described in detail [2]. As reported [2], patients were classified based on published criteria [37]. Both twins had to be living to participate in the study. Only samples of self-reported European ancestry were used for this study. The majority of twin pairs were female (*n* = 26, 96%), and approximately two thirds (*n* = 19, 70%) were monozygotic. The mean age of diagnosis was 43 years, and average disease duration from disease onset (first symptom attributable to SSc) was 8.8 years. We had 17 twin pairs with complete organ involvement data. Among these, the most frequent organ system involvement consisted of Raynaud’s phenomenon in 17 twin pairs (100%), joint or tendon in 15 (88%), gastrointestinal in 11 (65%), digital ulcers in 7 (41%), lung in 4 (24%), and renal involvement in 2 (12%). Among the 27 patients, the most common SSc-associated serum autoantibodies were anticentromere (*n* = 7, 26%), anti-RNA polymerases (n = 7, 26%), anti-topoisomerase I (*n* = 4, 15%), anti-U1 RNP (*n* = 3, 11%), and anti-U3 RNP (*n* = 3, 11%). For the disease subset analyses, 15 pairs with lcSSc and 9 pairs with dcSSc were used. Genomic DNA was extracted from whole blood from all 27 pairs of twins as previously described [2].

**Table 1:** Characteristics of the twin pairs discordant for SSc used for this analysis

Pair	Zygosity	Subtype	Gender	Autoantibody	Organ involvement
1	MZ	dcSSc	F/F	RNA pol	JT, GI, DU, RN
2	MZ	dcSSc	F/F	ATA	JT, GI, DU
3	MZ	dcSSc	F/F	RNA pol	JT
4	MZ	dcSSc	F/F	RNA pol	JT, LN, RN
5	MZ	dcSSc	F/F	U3	JT
6	DZ	dcSSc	F/F	RNA pol	JT, GI, DU
7	MZ	lcSSc	F/F	U1	GI
8	MZ	lcSSc	F/F	PL-7	JT, GI, LN
9	MZ	lcSSc	F/F	U3	JT, GI
10	DZ	lcSSc	F/F	ACA	JT, DU
11	DZ	dcSSc	F/F	ATA	JT, GI, DU, LN
12	MZ	dcSSc	F/F	Unknown	JT, GI, LN
13	MZ	dcSSc	F/F	RNA pol	N/A
14	DZ	lcSSc	F/F	ACA	JT
15	DZ	lcSSc	F/F	RNA pol	N/A
16	DZ	lcSSc	F/M	ATA	N/A
17	DZ	lcSSc	F/F	PM-Scl	JT, GI
18	DZ	lcSSc	F/F	U1	JT
19	MZ	lcSSc	F/F	ACA	JT, GI, DU
20	MZ	lcSSc	F/F	U3	GI, DU
21	MZ	lcSSc	F/F	ATA	N/A
22	MZ	lcSSc	M/M	RNA pol	N/A
23	MZ	lcSSc	F/F	ACA	N/A
24	MZ	lcSSc	F/F	ACA	N/A
25	MZ	lcSSc	F/F	ACA	N/A
26	MZ	lcSSc	F/F	ACA	N/A
27	MZ	lcSSc	F/F	U1	N/A

*MZ* monozygotic, *DZ* dizygotic, *F* female, *M* male, *ACA* anticentromere, *RNA pol* anti-RNA polymerases, *ATA* anti-topoisomerase I, *U1* anti-U1 RNP, *U3* anti-U3 RNP, *PL-7* anti-PL-7, *PM-Scl* anti-PM-Scl, *Unknown* autoantibodies did not recognize known autoantigens *JT* joint or tendon *GI* gastrointestinal, *DU* digital ulcers, *LN* lung, *RN* renal, *N/A* data not available

### Zygosity testing

Twin zygosity was initially assayed using DNA fingerprint analysis as we described [2]. In addition, zygosity was confirmed by the analysis of 11 short tandem repeat (STR) autosomal markers using the GenomeLab Human STR Primer Set kit on a CEQ8000 Genetic Analysis System (Beckman Coulter, Fullerton, CA) or 15 autosomal STR markers using the AmpFLSTR Identifiler PCR Amplification Kit on a 3500 Genetic Analyzer (Applied Biosystems, Foster City, CA). The manufacturer’s protocols were followed for both systems with one exception: separations on the 3500 Genetic Analyzer were performed with POP7 on a 50-cm array.

### DNA methylation assay and data analysis

Genomic DNA (1 μg) from each individual was treated with sodium bisulfite using the EZ 96-DNA methylation kit (Zymo Research, USA), following the manufacturer’s standard protocol. Genome-wide DNA methylation was assessed in the Genomics Research Core at the University of Pittsburgh using the Illumina Infinium HumanMethylation450 BeadChip (Illumina, USA), which interrogates over 485,500 CpG sites that cover 99% of RefSeq genes (including the promoter, 5′ UTR, first exon, gene body, and 3′UTR), as well as 96% of CpG islands and island shores. Arrays were processed using the manufacturer’s standard protocol. Location of individuals on arrays was randomized to minimize potential confounding (e.g., batch effects).

Sample files and expression IDAT files were imported into GenomeStudio Software v.1.9 (Illumina, USA) for primary evaluation of the data. This included initial quality control checks and calculating the relative methylation level of each interrogated cytosine, which is reported as a β-value given by the ratio of the normalized signal from the methylated probe to the sum of the normalized signals of the methylated and unmethylated probes. A negative β-value indicates hypomethylation (i.e., decreased methylation) in the affected SSc twins relative to the unaffected, while a positive β-value indicates hypermethylation (i.e., increased methylation) in the SSc twins relative to the unaffected twins. The data were observed for quality, and a cluster analysis was conducted, using the SNP content, to ensure twins were pairing correctly. Using GenomeStudio, it was noted that the data contained no large batch effects.

After initially inspecting the data with GenomeStudio, the data was opened with the R package ChAMP [38]. When loading the data, probes were dropped if they had a bead count less than 3, if the probed CpG was also an SNP, or if they did not meet a detection *p* value of 1 × 10^−5^ (detection p-value is the confidence that a given transcript is expressed above the background defined by negative probes). A total of 447,254 CpGs were used for analysis. The data were then normalized with the same ChAMP package using a BMIQ normalization method. MDS plots based on the 1000 most variable methylation sites were created as a result of the normalization process. These were examined for clustering, and it appeared that samples from individuals of differing ethnicities were clustering together, so some samples were removed to make a more homogenous group that clustered closely together. Singular value decomposition (SVD) was then applied to the matrix to obtain the most significant components of variation. These components were observed in a heat map showing the association between the principal components and the biological factors. To adjust for these batch effects, the ChAMP package employs “ComBat” which uses empirical Bayes methods to correct for technical variation. With the data normalized and batch effects adjusted for, the β-values were outputted to a table for analysis.

A paired *t* test was computed for each CpG site to test the null hypothesis that the mean difference of β-values for each set of twins is zero (*μ* = 0). Completing a matched analysis with the paired *t* test allows us to remove the confounding effects of chronological age, genetic background, ethnicity and admixture, sex, and similarity of the epigenome at birth. All data for monozygotic twins (*n* = 19) were analyzed first followed by a replication of that analysis with the data for dizygotic twins (*n* = 8). A meta-analysis of the two separate analyses was then performed using METAL [39] to get a single *p* value for each CpG site. False discovery rate (FDR) *p* values were then calculated for each site, and top results were evaluated. Since no differentially methylated cytosine was identified with FDR-corrected *p* < 0.05, unadjusted *p* values are reported. Only cytosines showing suggestive differential methylation (*p* < 10^−04^) in the meta-analysis between the affected and unaffected twin pairs are reported.

Monozygotic twins exhibit increased DNA methylation differences with age [40]. In order to address the effect of age on DNA methylation variation in this study, we cross-referenced our results (all cytosines with suggestive differential methylation (*p* < 10^−04^)) against the 490 and the 353 differentially methylated CpG sites associated with age reported by Bell et al. [41] and Horvath [42], respectively.

Despite the limited statistical power, an exploratory analysis was computed comparing all twins positive for each of the following clinical features: (1) lung involvement, (2) anticentromere autoantibodies (ACA), and (3) anti-RNA polymerases autoantibodies (anti-RNP), to the twins negative for these criteria. No CpGs met the threshold for suggestive differential methylation (*p* < 10^−04^) for any of these clinical features.

### Pathway analysis

Ingenuity Pathway Analysis (IPA) software (https://www.qiagenbioinformatics.com/products/ingenuity-pathway-analysis/) was used (release date 16 March 2016) to investigate the pathways and functions enriched with the molecules corresponding to the top differentially methylated genes. IPA uses an extensive database of functional interactions that are drawn from peer-reviewed publications and manually maintained. Core Analyses were performed using default settings to identify the top canonical pathways, diseases and biological functions, physiological systems, networks, and upstream regulators. For each comparison of the DNA methylation analyses, a total of 200 molecules (i.e., gene products) corresponding to the top differentially methylated cytosines were used as input for IPA Core Analyses. Specifically, the top 200 genes corresponding to the top differentially methylated cytosines were used as input into IPA’s Core Analysis; the products of these genes were used by IPA as molecules to predict, for example, downstream biological processes or diseases affected by the data, or upstream molecules which may be causing the observed changes in the data.

### Regulatory annotation

eFORGE v1.2 (http://eforge.cs.ucl.ac.uk/) [43] was used to identify if the associated CpGs were enriched in cell-specific regulatory elements, namely DNase I hypersensitive sites (DHSs) (markers of active regulatory regions) and loci with overlapping histone modifications (H3Kme1, H3Kme4, H3K9me3, H3K27me3, and H3K36me3) across available cell lines and tissues from the Roadmap Epigenomics Project, BLUEPRINT Epigenome, and ENCODE (Encyclopedia of DNA Elements) consortia data. In addition to predicting disease-relevant cell types, eFORGE can also assess cell-composition effects of heterogeneous tissues by detecting tissue-specific DHS and histone modification enrichment based on genomic location.

**Fig. 1:**
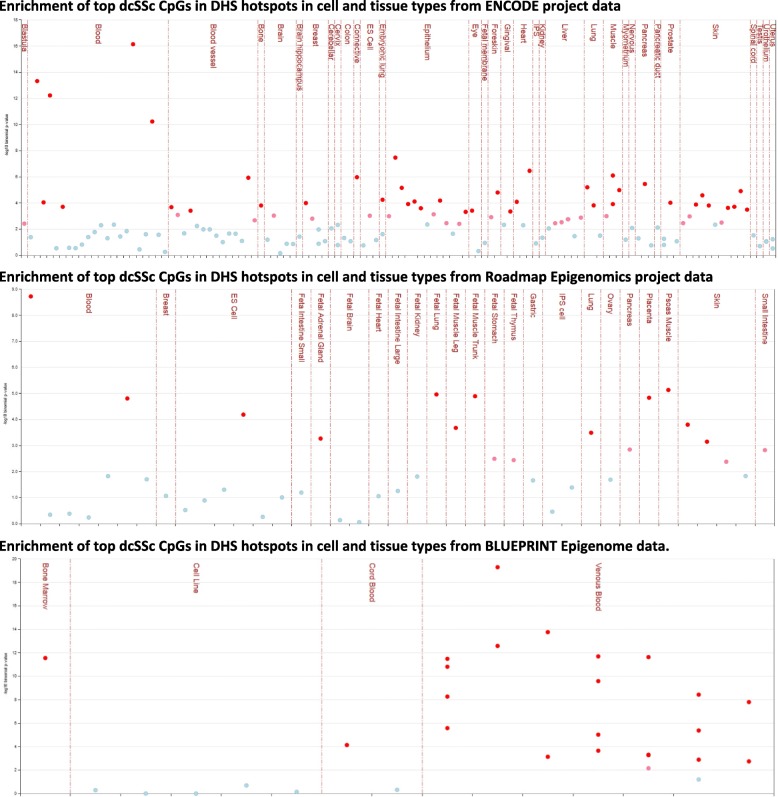
Enrichment of dcSSc differentially methylated CpGs in DNase I hypersensitive sites among various cell and tissue types using ENCODE, Roadmap Epigenomics, and BLUEPRINT Epigenome projects data. Statistically significant enrichment outside the 99.9th percentile (−log10 binomial *p* value ≥ 3.38) is colored red on the vertical axis. Upper panel shows a marked myeloid cell enrichment in ENCODE data, with strong epithelium, heart, muscle, blood vessel, and connective tissue signals. Middle panel shows a more general pattern of enrichment, strongest in blood, fetal tissues, and psoas muscle in the Roadmap Epigenomics data. Lower panel shows enrichment for inflammatory macrophages in the BLUEPRINT Epigenome data

**Fig. 2:**
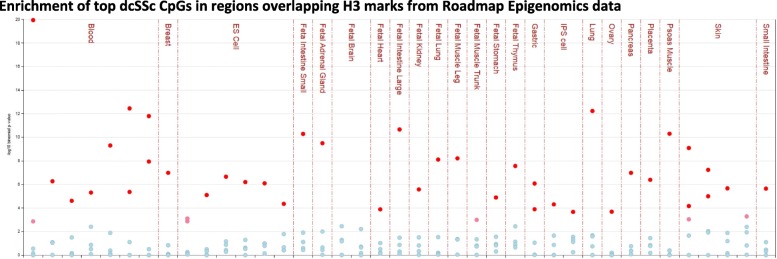
Enrichment of dcSSc differentially methylated CpGs in regions overlapping histone modifications in the Roadmap Epigenomics Project data. Statistically significant enrichment outside the 99.9th percentile (−log_10_ binomial *p* value ≥ 3.38) is colored red on the vertical axis. Panel shows marked enrichment for a histone modification representative of enhancers (H3K4me1) in blood cells (monocytes, hematopoietic stem cells, natural killer cells), fetal tissues (lung fibroblasts, large intestine, small intestine, adrenal gland, muscle leg, thymus), psoas muscle, and skin fibroblasts. Enrichment for a histone modification representative of polycomb-repressed regions (H3K27me3) was seen in hematopoietic stem cells

The differentially methylated cytosines (*p* < 10^−04^) in each disease subset (153 in lcSSc, 266 in dcSSc, and 155 in all twins) were entered as input of the eFORGE analysis (Additional file 2). Each set of CpGs was tested for enrichment for overlap with putative functional elements compared to matched background CpGs. The matched background is a set of the same number of CpGs as the test set, matched for gene relationship and CpG island relationship annotation. One thousand matched background sets were applied. The enrichment analysis was completed for different tissues, since functional elements may differ across tissues. Enrichment outside the 99.9th percentile (−log_10_ binomial *p*-value ≥ 3.38) was considered statistically significant (red in Additional file 2: Figure S2, Figs. 1 and 2).

## Results

### Differentially methylated sites in whole blood from twins discordant for SSc

We performed genome-wide DNA methylation analysis in whole blood from 27 twin pairs discordant for SSc (Table 1). Monozygotic twins (*n* = 19) were analyzed first, followed by a replication with the data for dizygotic twins (*n* = 8). This manuscript reports the results of the meta-analysis of this discovery and replication sets. A total of 155 cytosines showed suggestive differential methylation (*p* < 10^−04^) between the affected and unaffected twin pairs, most of which mapped to gene bodies (113, 73%) of 111 unique genes (Additional file 2: Table S1). We note that while a negative β-value (− 1 < β < 0) indicates hypomethylation (i.e., decreased methylation) in the affected SSc twins relative to the unaffected, a positive β-value (0 < β < 1) indicates hypermethylation (i.e., increased methylation) in the SSc relative to the unaffected twins. Results in monozygotic and dizygotic twin pairs were largely consistent (Additional file 2: Table S1). The levels of differential methylation between affected and unaffected twin were overall modest, with the largest difference observed in the *IFI44L* gene (β-value = − 0.12) (Additional file 1: Figure S1). Pathway analysis revealed a significant enrichment of molecules (i.e., gene products) involved in cancer, gastrointestinal disease, and organismal injury and abnormalities (Additional file 2: Table S2).

We also performed DNA methylation analyses in each disease subset. In the meta-analysis of 15 twin pairs discordant for lcSSc, 153 cytosines showed suggestive differential methylation (*p* < 10^−04^) between the affected and unaffected twin pairs, most of which mapped to gene bodies (117, 77%) of 115 distinct genes (Additional file 2: Table S3). The differences of methylation levels were modest (β < 0.10). Pathway analysis showed a significant enrichment of cancer, endocrine system disorders, gastrointestinal disease, and organismal injury and abnormalities (Additional file 2: Table S4). A total of 266 cytosines showed suggestive (meta-analysis *p* < 10^−04^) differential methylation levels in whole blood from the 9 pairs of twins discordant for dcSSc. The majority of these cytosines mapped to gene bodies (201, 76%) of 196 distinct genes (Additional file 2: Table S5). The largest differences in methylation levels were observed in the hypomethylated *IFI44L* (β = − 0.17) and *DHODH* (β = − 0.17) genes. The top molecules were enriched for cancer, gastrointestinal disease, and organismal injury and abnormalities (Additional file 2: Table S6). While there was virtually no overlap of molecules between subsets, with only 1% of common molecules (3/397), there was similar overall enrichment for genes in “cancer” and “gastrointestinal disease.”

Despite the limited statistical power, an exploratory case-case analysis was computed on the following clinical features: (1) lung involvement, (2) anticentromere autoantibodies (ACA), and (3) anti-RNA polymerases autoantibodies (anti-RNP). No CpGs met the threshold for suggestive differential methylation (*p* < 10^−04^) for any of these clinical features.

### Overlap of DNA methylation patterns with reported genetic association and DNA methylation studies

We assessed the overlap between the regions our study unveiled (meta-analysis *p* < 10^−04^) and over 40 regions with compelling evidence for genetic association with SSc [1]. The few regions of overlap include the *HLA* and *IRF5* (Additional file 2: Table S7). Since aging can influence DNA methylation variation, we also assessed the overlap between our results and the CpG sites whose methylation levels are strongly correlated with chronological age [42, 44]. Only one age-associated CpG (cg22432269) in the first exon of the *CYFIP1* gene showed concomitant evidence of hypomethylation in lcSSc (*p* = 3.06 × 10^−06^).

One genome-wide DNA methylation study has been reported in cultured dermal fibroblasts from SSc patients and controls [34]. As expected, given the different tissues profiled, the genes identified in each study are largely different. Of the 30 genes reported by Altorok et al [34] as common between dcSSc and lcSSc fibroblasts, only *CACNA1C* was also found among our top results (Additional file 2: Table S2). Additional file 2: Table S8 shows the six CpG sites common to both studies.

Since SSc and SLE are often considered related diseases, we also compared our DNA methylation findings in blood from SSc patients to cytosines reported as differentially methylated in blood (and blood cells) from SLE patients. These included 86 cytosines in naïve CD4+ T cells [45], 1082 CpGs in T cells, 264 CpGs in B cells, 168 CpGs in monocytes [15], 293 sites in neutrophils [46], 26,298 CpGs in PBMCs [47], and 44 cytosines differentially methylated in white blood cells from SLE patients [48]. A total of 76 CpGs differentially methylated in blood cells from both SSc and SLE patients are shown in Additional file 2: Table S9. Several cytosines were reported in multiple studies, notably those differentially methylated in the dcSSc subset, as well as all twins. CpG sites in the *IFI44L* and *RSAD2* genes were consistently hypomethylated in several blood cell types [15, 45–48] or hypermethylated in the case of *FNBP1* [15, 47]. In the dcSSc subset, CpG sites in *IRF5, INTS6, SULT1A1,* and *RPTOR* were also hypomethylated in multiple blood subsets [15, 46, 47]. These differentially methylated sites shared in blood cells across related autoimmune diseases suggest their role as potential susceptibility or epigenetic blood biomarkers of autoimmunity.

### Comparison of differential methylation to differential gene expression patterns

To explore the downstream effects of the differentially methylated CpG sites (meta-analysis *p* < 10^−04^), the genes corresponding to these CpGs were compared to available data from published global gene expression profiling studies conducted in blood and its cellular subsets from SSc patients and healthy controls. A total of 1907 unique differentially expressed genes were compiled from 8 studies with publicly available results [49–56]. As shown in Table 2, 27 genes with differentially methylated cytosines (in Additional file 2: Tables S1, S3, and S5) have also been reported as differentially expressed in SSc patients. Consistent with the known complex relationships between DNA methylation and gene expression [4–11], for some genes, the relationship between DNA methylation and gene expression was inverse or negative (i.e., increased methylation with decreased gene expression), while for others, it was direct or positive (i.e., increased methylation results in increased gene expression).

**Table 2:** Genes showing concomitant evidence for differential DNA methylation and gene expression in blood from SSc patients

		DNA methylation													Reported gene expression	
Gene	Location	This study						Other reports							Expression	Refs
		CpG	Gene location	CpG island location	All	lcSSc	dcSSc	SLE						SSc		
								Naïve T cells [17, 23]	T cells [15]	B cells [15]	Mono- cytes [15]	Neutro- phils [45, 8]	WBC [47]	skin fibrob [34]		
RPS6KA1	1p36.11	cg24585377	Body	S_Shore			down-		*down-*						up-	[53]
IFI44L	1p31.1	cg03607951	TSS1500	-	down-		down-	down-	down-	down-	down-	down-	down-		up-	[49, 50, 52, 29, 27]
RSAD2	2p25.2	cg15346781	TSS1500	-	down-			*down-*	down-	down-	*down-*		*up-*		up-	[49, 52, 27]
LPIN1	2p25.1	cg02069619	TSS1500	N_Shore			down-								down-	[49]
LPP	3q27.3	cg05685023	TSS1500	N_Shore			down-								down-	[53]
RPL37	5p13.1	cg08754067	Body	N_Shelf	up-										down-	[49]
HMGCR	5q13.3	cg26399773	5'UTR	Island		up-									up-	[50]
TNXB	6p21.33	cg06580770	Body	N_Shore	up-									down-	down-	[53]
		cg02673305	5'UTR	S_Shore		down-								down-	down-	[53]
KCNQ1	11p15.5	cg07824422	Body	Island			down-								up-	[53]
CD248	11q13.2	cg00350296	TSS1500	S_Shore	down-										up-	[48]
CACNA2D4	12p13.33	cg19069360	Body	-			down-								up-	[50]
TNFRSF1A	12p13.31	cg26254667	TSS1500	-			up-		*up-*						up-	[50, 53, 24]
WDFY2	13q14.3	cg08029014	Body	-	up-										up-	[50]
ITPK1	14q32.12	cg25095171	Body	N_Shelf		down-									up-	[53]
CRIP2	14q32.33	cg15532667	TSS1500	N_Shore		down-									down-	[49]
CYFIP1	15q11.2	cg22432269^*^	1stExon	Island		down-									up-	[50]
SQRDL	15q21.1	cg01626885	5'UTR	-			down-								up-	[50]
TLE3	15q23	cg01666796	Body	-	up-										up-	[48]
		cg12349571	Body	-	up-										up-	[48]
ALOX15	17p13.2	cg06222638	Body	Island	down-										down-	[53]
STAT3	17q21.2	cg24312520	Body	-			up-		*up-*	*up-*					up-	[48, 50, 26]
CBX4	17q25.3	cg00483030	Body	N_Shore			down-								up-	[52]
PTPRS	19p13.3	cg08857677	TSS1500	S_Shore		down-									up-	[53]
TYROBP	19q13.12	cg17925829	TSS200	-			down-								up-	[52, 53, 27]
RASGRP4	19q13.2	cg24376214	TSS1500	-			up-								up-	[50]
RIN2	20p11.23	cg12049875	Body	Island			down-								up-	[50]
PRIC285	20q13.33	cg01458054	Body	N_Shore	up-			*up-*	*up- or down-*	*up- or down-*	*down-*		*up-*		up-	[49]
CERK	22q13.31	cg05602642	3'UTR	N_Shore		down-									down-	[49]

Up- and down- refer to hypo- or hypermethylation, or over- or underexpression, respectively. Italics denotes methylation of different CpG sites in the gene. TSS1500 (TSS200), within 1500 bps (200 bps) from transcription start site; 5′UTR (3'UTR), 5′ (3') untranslated region. WBC: white blood cells. Skin fibrob: skin fibroblasts. Refs: references. *Age-associated CpG [42].

Eight noteworthy candidates include *IFI44L*, where cg03607951 in the transcription start site was hypomethylated in all twins and showed the largest difference in methylation levels in dcSSc. This gene is overexpressed in blood in SSc patients [50, 51, 53] and hypomethylated in multiple blood SLE subsets [15, 45, 46, 48]. The *TLE3* gene showed two CpGs in the gene body (cg01666796, cg12349571) with consistent hypermethylation in all affected twins and is also overexpressed in PBMCs in SSc-PAH patients [49]. A CpG (cg22432269) in the first exon of the *CYFIP1* gene showed the most significant hypomethylation in lcSSc concomitant with underexpression in PBMCs from lcSSc patients [51]. A CpG site (cg06580770) in the body of *TNXB* is hypermethylated in blood from SSc and concomitantly underexpressed in SSc patients [54]. Hypomethylated cg15346781 in the transcription start site of the *RSAD2* gene is overexpressed in SSc [50, 53] and hypomethylated in T and B cells from SLE patients [15]. Cg24312520 in the gene body of *STAT3* was hypermethylated in dcSSc and overexpressed in PBMC from lcSSc and SSc-PAH patients [49, 51]. A first exon cytosine (cg25330422) was reported as hypermethylated in blood cell subsets from SLE patients [15]. The transcription start site *TNFRSF1A* was both hypermethylated in dcSSc (cg26254667) and overexpressed in SSc and lcSSc [51, 54]. Other gene body CpG sites (cg08418872, cg23752651) have also been reported as hypermethylated in SLE patients [15]. Lastly, cg17925829 in the transcription start site of the *TYROBP* gene was hypomethylated and the gene overexpressed in SSc [53, 54].

### Regulatory annotation

To provide a broader biological interpretation of the DNA methylation results and better understand the functional role underlying the disease-associated CpG sites, we assessed whether these SSc-associated CpGs reside within regulatory regions across the genome in diverse tissues and cell types assayed in the ENCODE, Roadmap Epigenomics, and BLUEPRINT Epigenome Project datasets. The CpGs associated with SSc in all twins showed only a modest enrichment of H3K27me3, a mark of inactive genes, in primary B cells (Additional file 1: Figure S2 and Additional file 2: Table S10). The CpGs associated with lcSSc did not show enrichment of either DHSs or any histone mark in any tissue. In contrast, the dcSSc-associated CpGs showed robust enrichment in DHSs across multiple tissues and cell types (Fig. 1; Additional file 2: Table S11). In the ENCODE data, the strongest enrichment was in multiple blood cell lines, predominately myeloid cells, but also the epithelium, heart, muscle, blood vessel, and connective tissue (Fig. 1, top panel; Additional file 2: Table S11). In the Roadmap data, the greatest enrichment of DHS was in the blood, fetal tissues, and psoas muscle (Fig. 1, middle panel; Additional file 2: Table S11). In the hematopoietic primary cells of the BLUEPRINT project, inflammatory macrophages showed strong enrichment of DHSs (Fig. 1, bottom panel; Additional file 2: Table S11). This data provides evidence supporting the notion that CpGs identified in blood are also situated in known active regulatory regions in not only blood, but also other tissues and cell types. Overlapping with H3 histone methylation from the Roadmap Project revealed that the dcSSc-associated CpGs are strongly enriched for H3K4me1 marks, which are indicative of poised enhancers, across numerous tissues and cell types, most strongly in the blood, fetal tissues, psoas muscle, and skin (Fig. 2, Additional file 2: Table S11). An enrichment of H3K27me3, a mark associated with inactive gene promoters, was also detected in primary hematopoietic stem cells (Fig. 2, Additional file 2: Table S11).

Collectively, this regulatory annotation data shows that, unlike the lcSSc-associated CpGs, many of the dcSSc-associated CpGs reside within DHS and multiple histone marks. This evidence of enrichment of regulatory regions supports their potential role in causal downstream effects on disease presentation.

## Discussion

This study used a genome-wide integrative approach to identify differential DNA methylation in whole blood from twin pairs discordant for SSc. In addition to being the largest epigenomic study conducted in SSc to date, the unique study design minimizes confounding due to genetic heterogeneity and age- and early-life environmental effects by using disease-discordant twins [35, 36]. As expected, given the sample size, we did not detect genome-wide significant differences in mean DNA methylation associated with SSc, which is largely consistent with other complex disease epigenomic twin studies [12, 57, 58]. The results revealed distinct DNA methylation patterns in SSc and its clinical disease subsets. The negligible overlap of molecules shared between the lcSSc and dcSSc subsets supports distinct epigenetic architectures in each disease subset. Despite clearly distinct blood methylation profiles, an enrichment of genes in “cancer” and “gastrointestinal disease” was observed in both dcSSc and lcSSc, although driven by different molecules. These results are consistent with the previously reported minimal common differentially methylated cytosines between lcSSc and dcSSc subsets in skin fibroblasts [34]. In addition, our analyses revealed negligible overlap between the methylation patterns in whole blood and those previously reported in skin fibroblasts [34]. Thus, although SSc is commonly considered a single disease, these results confirm others suggesting that SSc is a family of diseases with distinctly different subtypes.

The precise relationships between DNA methylation and gene expression are complex and poorly understood [4–11]. While DNA methylation at regulatory elements shows a negative correlation with transcription, the opposite has been observed at intragenic regions [5], illustrating that complex regulatory mechanisms that are dependent on the tissue and genomic architecture underlie the correlation between DNA methylation and gene expression. It is also possible that the low correlation between DNA methylation and gene expression levels may reflect high fluctuation of RNA levels, which can change from 1 h to the next [59]. In order to provide insights into the potential functional consequences of the methylation patterns observed, we compared our results to those of global gene expression profiling assays conducted in blood and its cellular subsets from SSc patients and healthy controls. This study unveiled several novel genes epigenetically dysregulated with reported changes in gene expression in blood from SSc patients. Most of these genes are involved in immune processes.

*IFI44L*, an interferon gene involved in defense response to viruses, is overexpressed in SSc blood tissues [50, 51, 53]. The CpG site unveiled in our study shows consistent hypomethylation in multiple blood cell subsets from SLE [15, 45–48, 60] and Sjögren’s syndrome patients [61, 62]. Since SSc and SLE are often considered as sister diseases, reported DNA methylation similarities are not unexpected [63]. The consistent hypomethylation of *IFI44L* in blood from patients with several autoimmune diseases, together with its overexpression, corroborates the validity of our finding and suggests that differential methylation of IFI44L may serve as shared biomarker across these diseases.

Both *RSAD2* and *TYROBP* showed hypomethylation and overexpression in SSc blood [50, 53, 54]. Both play roles in immune response, including type I IFN signaling pathway (RSAD2) and innate immunity (TYROBP). *RSAD2* is consistently hypomethylated in blood cells [15, 47]. Demethylation of the *TYROBP* gene is associated with a subset of T cells that accumulates and is associated with aging [64]. An age-associated CpG [42] in *CYFIP1*, a regulator of translation and cytoskeletal dynamics, showed hypomethylation with underexpression in SSc blood [51]. It is interesting to note the variation in methylation levels at sites associated with aging, as premature activation of aging-associated molecular mechanisms is emerging as an important contributor to the autoimmune, vascular, and fibrotic pathogenesis of SSc [65]. Our findings, in conjunction with these reports, further lend support for the role of the innate immune response in the pathogenesis and/or progression of diseases such as SSc and a parallel between SSc and premature aging.

Differential methylation of several genes has been reported as associated with cancer [66–69]. These include *TNFRSF1A*, which plays a role in cell survival, apoptosis, and inflammation and was both hypermethylated and overexpressed in SSc blood [51, 54]. *TLE3* was also hypermethylated and overexpressed in SSc blood [49]. This gene product functions in the Notch signaling pathway to regulate the determination of cell fate during development. *STAT3*, a transcription activator with roles in many cellular processes such as cell growth, apoptosis, and response to cytokines and growth factors, showed hypermethylation and overexpression in SSc blood [49, 51]. *TNXB,* which was hypomethylated in skin fibroblasts from dcSSc [34], was hypermethylated in our study and concomitantly underexpressed in blood from SSc patients [54]. This gene localizes to the MHC class III region and encodes a member of the tenascin family of extracellular matrix glycoproteins. It is involved in actin cytoskeleton organization, cell adhesion, and collagen fibril organization.

To aid in result interpretation, regulatory annotation of the top differentially methylated cytosines was conducted to predict disease-relevant cell types. Differential DNA methylations in regulatory regions such as DHS and histone marks have been associated with functional consequences [4, 70]. We observed an enrichment of regulatory regions in the dcSSc subset that pointed to blood myeloid cells as the most highly enriched cell types, indicating a tendency for cell-composition-corrected dcSSc-associated DNA methylation changes to co-locate with myeloid cell DHSs and H3K4me1 marks (representative of enhancers). This contrasts with an enrichment in DHSs specific to T cells that was reported using cytosines differentially methylated in CD4+ T cell studies of SLE and Sjögren’s syndrome [43]. This enrichment of methylated cytosines in regulatory regions in myeloid cells might underlie a dysregulation of these cells in dcSSc. Indeed, both monocytes and macrophages (cell types with the strongest enrichment) play a critical role in fibrosis [71]. The number of circulating monocytes is increased in SSc [72] and correlates with disease progression and severity [73, 74]. The changes in methylation detected in dcSSc are thus impacting the function of regulatory elements in cell types with critical functions in fibrosis. Since these inflammatory cells are dysregulated in SSc, and DNA methylation changes can affect regulatory mechanisms, our findings suggest that DNA methylation might be a potential avenue to reverse their altered phenotype.

This study has a number of limitations. Despite the value of the twin-pair study design for epigenomic studies, our unique samples of middle-aged, European ancestry, largely female twin pairs are not representative of the general population. Thus, our results might not be generalizable to all patients. Further replication studies are warranted for the validation, justification, and generalization of our results. Another limitation is the lack of available RNA from the same samples to assess the functional effects of the variation in DNA methylation. In an attempt to circumvent this limitation, we performed in silico integration with reported differentially expressed genes for functional validation of our results. Documenting that differentially methylated sites in our twin data also correspond to differences in gene expression in independent SSc samples forms corroborating evidence across genomic processes and cohorts. A further limitation is the lack of tissue specificity. We explored this issue by performing regulatory annotation of our results, but future work is needed to dissect the tissue specificity of epigenetic modifications in SSc. We cannot exclude the possibility that the differences between the disease subsets and enrichment of myeloid-related cells in dcSSc are driven by confounding cell-composition effects instead of true cell type-specific effects. However, whole blood lymphocytes are proportionally more abundant than monocytes, suggesting that the strong bias towards monocytes and macrophages is a cell type-specific effect. Multiple differentially methylated cytosines in our study were also found to be differentially methylated in a single blood cell type in SLE, suggesting that the associations we detected are not likely to be due to confounding by blood cell heterogeneity. These include, among others, loci in the *IFI44L*, *RSAD2*, *IRF5*, and *RPTOR* genes [46]. In spite of these limitations, these findings identify novel genomic regions in SSc in a unique cohort of discordant twins and highlight candidate genes for further research.

## Conclusions

We identified multiple DNA methylation loci associated with SSc, including sites with concomitant evidence of altered methylation in blood cells of lupus patients and genes with concomitant evidence of differential expression in blood cells from SSc patients. Although this cross-sectional study cannot separate causality from response to disease, it identifies epigenetically modified genes and pathways that are important in SSc.

Our study hence provides support for using blood cells as a useful accessible tissue for epigenetic biomarker discovery. Our results show that DNA methylation sites in dcSSc patients are enriched for regulatory regions in cell types with key roles in fibrosis, implicating DNA methylation as a modulator of cell functionality. Coupled with the observation that dcSSc and lcSSc are epigenetically distinct disease subtypes, this suggests that the cellular dysfunction observed in dcSSc is, at least partially, due to an epigenetic dysregulation of myeloid cell types. Further, this suggests the possibility of using epigenetic regulation of cell functionality to prevent dysfunction or restore their balance in SSc. Regardless of causality, blood-based biomarkers have the potential to improve risk prediction and help guide treatment decisions. Our findings provide a foundation for further research to determine if the differentially methylated functional loci represent attractive targets for the treatment or prevention of autoimmune- and/or fibrotic-related diseases.

## Additional files

Additional file 1: **Figure S1.** Visualization of absolute weighted β-values in whole blood from twin pairs discordant for SSc in the UCSC genome browser showing differential methylation of the IFI44L gene. Figure S2. Enrichment of SSc differentially methylated CpGs in regions overlapping histone modifications in the Roadmap Epigenomics Project data. (DOC 536 kb)

Additional file 2: **Table S1.** Most significant differentially methylated cytosines in whole blood from twins discordant for SSc. **Table S2.** Most significant canonical pathways, upstream regulators, and diseases and biological functions in differentially methylated genes in whole blood from all twin pairs discordant for SSc. **Table S3.** Most significant differentially methylated cytosines in whole blood from twins discordant for lcSSc. **Table S4.** Most significant canonical pathways, upstream regulators, and diseases and biological functions in differentially methylated genes in whole blood from twin pairs discordant for lcSSc. **Table S5.** Most significant differentially methylated cytosines in whole blood from twins discordant for dcSSc. **Table S6.** Most significant canonical pathways, upstream regulators, and diseases and biological functions in differentially methylated genes in whole blood from twin pairs discordant for dcSSc. **Table S7.** Reported SSc-associated gene regions with differentially methylated CpGs in SSc subsets. **Table S8.** Differentially methylated CpG sites common to this study and to the report by Altorok et al. (2015). **Table S9.** Cytosines differentially methylated in this study that are also reported as differentially methylated in blood from SLE patients. **Table S10.** Most significant enrichment of top SSc CpGs overlapping cell type-specific regulatory elements. Table S11. Most significant enrichment of top dcSSc CpGs overlapping cell type-specific regulatory elements. (DOC 913 kb)

## Abbreviations

dcSSc: Diffuse cutaneous SSc
DHSs: DNase I hypersensitive sites
lcSSc: Limited cutaneous SSc
SLE: Systemic lupus erythematosus
SSc: Systemic sclerosis
