# Supplementary material for: Integrative analysis of DNA methylation in discordant twins unveils distinct architectures of systemic sclerosis subsets
Source: Clin Epigenetics. 2019 Apr 4;11:58. doi: 10.1186/s13148-019-0652-y (PMC6449959; doi:10.1186/s13148-019-0652-y)
Supplement: Supplementary file 1 — Figure S1. Visualization of absolute weighted β-values in whole blood from twin pairs discordant for SSc in the UCSC genome browser showing differential methylation of the IFI44L gene. Figure S2. Enrichment of SSc differentially methylated CpGs in regions overlapping histone modifications in the Roadmap Epigenomics Project data. (DOC 536 kb) [file 13148_2019_652_MOESM1_ESM.doc]

Additional files for

**Ramos *et al*. Integrative analysis of DNA methylation in discordant twins unveils distinct architectures of systemic sclerosis subsets**

Table of Contents

Additional file 1: Supplementary Figures 2

Additional file 2: Supplementary Tables 4

References 30

**Supplementary Figures**

|  |
| --- |
| **Figure S1**. Visualization of absolute weighted β-values in whole blood from twin pairs discordant for SSc in the UCSC genome browser showing differential methylation of the IFI44L gene. Orange bar below the differentially methylated sites in the IFI44L promoter marks the transcription factor binding sites for STAT1, SMARCA4, RBL2, and TAL1 according to the ORegAnno database available as a UCSC track. |

| **Enrichment of top SSc CpGs in regions overlapping H3 marks from Roadmap Epigenomics data** |
| --- |
| 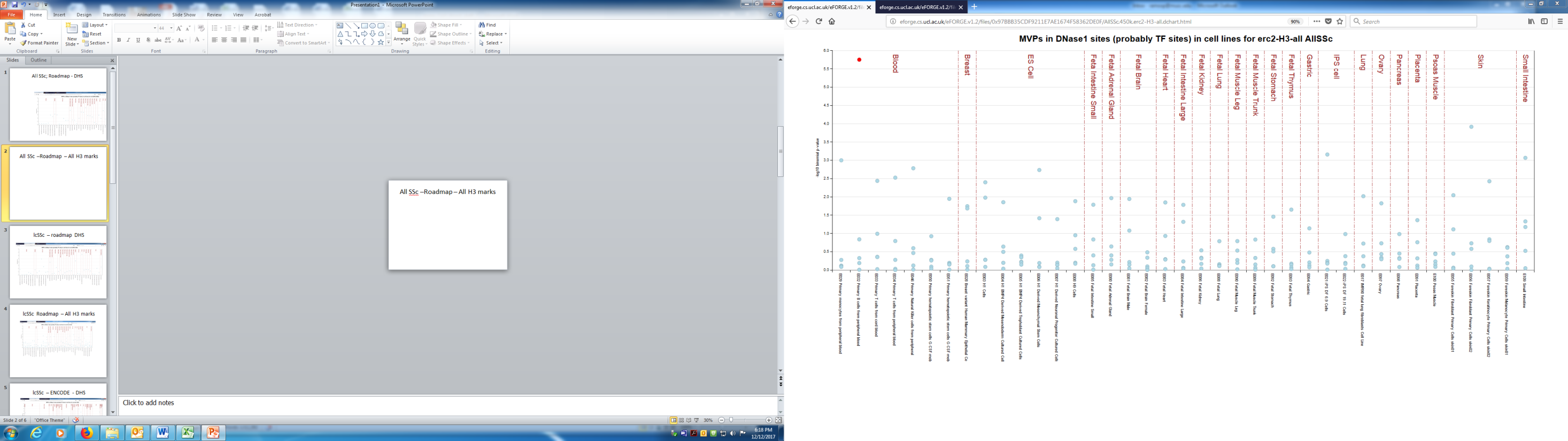 |
|  |
| **Figure S2.**  **Enrichment of SSc differentially methylated CpGs in regions overlapping histone modifications in the Roadmap Epigenomics Project data.**  Statistically significant enrichment outside the 99.9th percentile (−log_10_ binomial P-value ≥ 3.38) is colored red (vertical axis). Panel shows a modest enrichment for a histone modification representative of polycomb-repressed regions (H3K27me3) in primary B cells from peripheral blood. |
