# Supplementary material for: Integrative analysis of DNA methylation in discordant twins unveils distinct architectures of systemic sclerosis subsets
Source: Clin Epigenetics. 2019 Apr 4;11:58. doi: 10.1186/s13148-019-0652-y (PMC6449959; doi:10.1186/s13148-019-0652-y)
Supplement: Supplementary file 2 — Table S1. Most significant differentially methylated cytosines in whole blood from twins discordant for SSc. Table S2. Most significant canonical pathways, upstream regulators, and diseases and biological functions in differentially methylated genes in whole blood from all twin pairs discordant for SSc. Table S3. Most significant differentially methylated cytosines in whole blood from twins discordant for lcSSc. Table S4. Most significant canonical pathways, upstream regulators, and diseases and biological functions in differentially methylated genes in whole blood from twin pairs discordant for lcSSc. Table S5. Most significant differentially methylated cytosines in whole blood from twins discordant for dcSSc. Table S6. Most significant canonical pathways, upstream regulators, and diseases and biological functions in differentially methylated genes in whole blood from twin pairs discordant for dcSSc. Table S7. Reported SSc-associated gene regions with differentially methylated CpGs in SSc subsets. Table S8. Differentially methylated CpG sites common to this study and to the report by Altorok et al. (2015). Table S9. Cytosines differentially methylated in this study that are also reported as differentially methylated in blood from SLE patients. Table S10. Most significant enrichment of top SSc CpGs overlapping cell type-specific regulatory elements. Table S11. Most significant enrichment of top dcSSc CpGs overlapping cell type-specific regulatory elements. (DOC 913 kb) [file 13148_2019_652_MOESM2_ESM.doc]

**Supplementary Tables**

| **Table S1. Most significant differentially methylated cytosines in whole blood from twins discordant for SSc.** | | | | | | | | | | | |
| --- | --- | --- | --- | --- | --- | --- | --- | --- | --- | --- | --- |
| **CpG** | **Chr** | **Position (bp)** | **RefGene** | **MZ P-value** | **DZ P-value** | **Dir** | **MZ Beta** | **DZ Beta** | **W Beta** | **Meta**  **P-value** |  |
| cg00062072 | 1 | 1157684 | SDF4 | 4.28E-03 | 5.56E-03 | ++ | 0.02 | 0.02 | 0.02 | 9.40E-05 |  |
| cg06970772 | 1 | 3272662 | PRDM16 | 1.13E-01 | 8.74E-07 | ++ | 0.02 | 0.06 | 0.03 | 6.17E-05 |  |
| cg10206452 | 1 | 15442301 | KIAA1026;C1orf126 | 6.63E-03 | 5.58E-05 | ++ | 0.02 | 0.02 | 0.02 | 7.79E-06 |  |
| cg09554876 | 1 | 49224825 | BEND5;AGBL4 | 1.54E-02 | 6.35E-04 | ++ | 0.01 | 0.03 | 0.02 | 9.94E-05 |  |
| cg07790169 | 1 | 49242932 | BEND5;AGBL4 | 6.99E-04 | 1.67E-02 | -- | -0.01 | -0.01 | -0.01 | 3.38E-05 |  |
| cg03607951 | 1 | 79085586 | IFI44L | 4.44E-03 | 4.85E-03 | -- | -0.09 | -0.20 | -0.12 | 8.85E-05 |  |
| cg18800235 | 1 | 94486895 | ABCA4 | 1.02E-02 | 2.72E-04 | ++ | 0.02 | 0.05 | 0.03 | 3.53E-05 |  |
| cg06900257 | 1 | 167409054 | CD247 | 4.28E-03 | 8.44E-04 | -- | -0.04 | -0.08 | -0.05 | 2.51E-05 |  |
| cg24083324 | 1 | 214162604 | PROX1 | 2.35E-04 | 7.84E-02 | -- | -0.01 | -0.01 | -0.01 | 5.26E-05 |  |
| cg05773194 | 1 | 226844154 | ITPKB | 4.18E-03 | 4.13E-03 | -- | -0.02 | -0.07 | -0.04 | 7.38E-05 |  |
| cg15346781 | 2 | 7017571 | RSAD2 | 2.19E-02 | 2.65E-04 | -- | -0.03 | -0.11 | -0.06 | 9.29E-05 |  |
| cg24343835 | 2 | 56216234 | MIR216A | 6.20E-04 | 1.93E-02 | ++ | 0.03 | 0.05 | 0.03 | 3.40E-05 |  |
| cg23678058 | 2 | 60722313 | BCL11A | 3.82E-04 | 4.24E-03 | ++ | 0.03 | 0.04 | 0.03 | 5.71E-06 |  |
| cg17945976 | 2 | 66667433 | MEIS1 | 2.26E-03 | 1.05E-03 | -- | -0.01 | -0.01 | -0.01 | 1.39E-05 |  |
| cg14102186 | 2 | 85364676 | TCF7L1 | 1.93E-03 | 8.05E-03 | ++ | 0.02 | 0.03 | 0.02 | 5.27E-05 |  |
| cg12208353 | 2 | 152527013 | NEB | 1.44E-04 | 8.02E-03 | ++ | 0.02 | 0.04 | 0.03 | 3.62E-06 |  |
| cg05364508 | 2 | 203242417 | BMPR2 | 4.41E-02 | 5.06E-05 | -- | -0.01 | -0.03 | -0.02 | 9.84E-05 |  |
| cg13859511 | 2 | 219749205 | WNT10A | 3.77E-04 | 8.09E-02 | -- | -0.01 | -0.01 | -0.01 | 8.39E-05 |  |
| cg14692768 | 2 | 219925688 | IHH | 2.67E-04 | 2.77E-02 | -- | -0.02 | -0.01 | -0.02 | 2.08E-05 |  |
| cg07058918 | 2 | 233320486 | ALPI | 2.72E-02 | 8.02E-05 | ++ | 0.03 | 0.05 | 0.04 | 6.36E-05 |  |
| cg21368566 | 2 | 236568913 | AGAP1 | 2.49E-05 | 2.59E-02 | ++ | 0.02 | 0.02 | 0.02 | 2.04E-06 |  |
| cg17619311 | 3 | 42947565 | ZNF662 | 1.63E-03 | 2.45E-03 | -- | -0.05 | -0.06 | -0.06 | 1.77E-05 |  |
| cg23430388 | 3 | 63429828 | SYNPR | 3.03E-03 | 6.77E-03 | ++ | 0.04 | 0.08 | 0.05 | 7.45E-05 |  |
| cg12193622 | 3 | 101567865 | NFKBIZ | 4.20E-04 | 4.83E-02 | -- | 0.00 | -0.01 | 0.00 | 5.49E-05 |  |
| cg22838882 | 3 | 138952118 | PISRT1 | 5.99E-03 | 2.76E-03 | ++ | 0.01 | 0.02 | 0.02 | 8.32E-05 |  |
| cg23351724 | 3 | 151177604 | IGSF10 | 2.33E-03 | 6.77E-03 | ++ | 0.01 | 0.02 | 0.01 | 5.63E-05 |  |
| cg22126211 | 3 | 164914600 | SLITRK3 | 3.11E-03 | 7.58E-03 | -- | -0.03 | -0.05 | -0.03 | 8.35E-05 |  |
| cg12226453 | 4 | 964011 | DGKQ | 7.49E-02 | 6.23E-06 | ++ | 0.02 | 0.06 | 0.03 | 7.70E-05 |  |
| cg12044923 | 4 | 5207312 | STK32B | 7.34E-02 | 8.18E-07 | ++ | 0.03 | 0.03 | 0.03 | 2.84E-05 |  |
| cg19143170 | 4 | 81303914 | C4orf22 | 2.96E-03 | 9.38E-03 | ++ | 0.03 | 0.04 | 0.03 | 9.34E-05 |  |
| cg05242526 | 4 | 86396103 | ARHGAP24 | 7.55E-03 | 6.62E-04 | -- | -0.01 | -0.02 | -0.01 | 4.23E-05 |  |
| cg10857558 | 4 | 90035421 | TIGD2 | 1.83E-03 | 1.30E-02 | ++ | 0.03 | 0.04 | 0.03 | 7.28E-05 |  |
| cg08754067 | 5 | 40833236 | SNORD72;RPL37 | 1.44E-04 | 1.14E-01 | ++ | 0.04 | 0.03 | 0.04 | 5.11E-05 |  |
| cg16181978 | 5 | 68531229 | CDK7 | 2.46E-03 | 1.28E-02 | -- | -0.01 | -0.01 | -0.01 | 9.79E-05 |  |
| cg02124184 | 5 | 72950691 | RGNEF | 3.91E-03 | 1.60E-03 | ++ | 0.02 | 0.03 | 0.02 | 3.50E-05 |  |
| cg26068911 | 5 | 89967277 | GPR98 | 2.18E-04 | 1.19E-02 | ++ | 0.03 | 0.04 | 0.04 | 7.78E-06 |  |
| cg11853830 | 5 | 92914036 | FLJ42709 | 9.95E-03 | 1.33E-03 | -- | -0.04 | -0.08 | -0.05 | 9.27E-05 |  |
| cg12699327 | 5 | 135170782 | LOC153328 | 8.39E-05 | 5.47E-02 | -- | -0.01 | -0.01 | -0.01 | 1.39E-05 |  |
| cg08490107 | 5 | 171774272 | SH3PXD2B | 6.21E-02 | 7.25E-06 | ++ | 0.02 | 0.04 | 0.03 | 6.15E-05 |  |
| cg09016806 | 6 | 30120365 | TRIM10 | 1.17E-04 | 2.13E-01 | ++ | 0.01 | 0.01 | 0.01 | 9.21E-05 |  |
| cg23491756 | 6 | 31438151 | HCG26 | 5.19E-02 | 1.82E-05 | -- | 0.00 | -0.01 | -0.01 | 7.36E-05 |  |
| cg06580770 | 6 | 32054790 | TNXB | 7.65E-02 | 5.53E-06 | ++ | 0.02 | 0.04 | 0.03 | 7.52E-05 |  |
| cg12583553 | 6 | 33242487 | RPS18 | 9.77E-03 | 6.95E-04 | ++ | 0.03 | 0.06 | 0.04 | 5.98E-05 |  |
| cg07541160 | 6 | 36985879 | FGD2 | 1.35E-02 | 1.80E-04 | ++ | 0.02 | 0.03 | 0.02 | 3.94E-05 |  |
| cg00255719 | 6 | 71377386 | SMAP1 | 1.40E-04 | 1.52E-01 | -- | 0.00 | 0.00 | 0.00 | 7.04E-05 |  |
| cg02285263 | 6 | 152129749 | ESR1 | 9.39E-04 | 3.15E-02 | -- | 0.00 | -0.01 | -0.01 | 7.94E-05 |  |
| cg10601168 | 6 | 154831192 | CNKSR3 | 9.19E-04 | 3.58E-02 | -- | -0.01 | -0.01 | -0.01 | 8.75E-05 |  |
| cg05867499 | 7 | 4848814 | RADIL | 7.97E-04 | 3.17E-02 | -- | -0.02 | -0.09 | -0.04 | 6.81E-05 |  |
| cg14780416 | 7 | 27209338 | MIR196B | 3.39E-05 | 8.26E-02 | -- | -0.01 | -0.01 | -0.01 | 9.76E-06 |  |
| cg25037274 | 7 | 29872894 | WIPF3 | 8.10E-03 | 1.79E-03 | ++ | 0.01 | 0.03 | 0.02 | 8.81E-05 |  |
| cg12300292 | 7 | 72847862 | FZD9 | 3.45E-03 | 7.41E-03 | ++ | 0.01 | 0.02 | 0.01 | 9.19E-05 |  |
| cg08637123 | 7 | 138764793 | ZC3HAV1 | 7.11E-04 | 6.70E-03 | ++ | 0.01 | 0.01 | 0.01 | 1.59E-05 |  |
| cg06887471 | 7 | 149157803 | ZNF777 | 5.05E-04 | 8.58E-03 | -- | -0.01 | -0.01 | -0.01 | 1.37E-05 |  |
| cg20665259 | 7 | 150659402 | KCNH2 | 3.66E-03 | 6.10E-03 | ++ | 0.04 | 0.04 | 0.04 | 8.48E-05 |  |
| cg20915447 | 8 | 1711433 | CLN8 | 7.97E-04 | 1.63E-02 | -- | -0.01 | -0.01 | -0.01 | 3.77E-05 |  |
| cg09758595 | 8 | 11141996 | MTMR9 | 1.19E-05 | 3.72E-01 | ++ | 0.00 | 0.00 | 0.00 | 3.19E-05 |  |
| cg24909309 | 8 | 38387291 | C8orf86 | 1.18E-02 | 7.91E-04 | ++ | 0.02 | 0.04 | 0.03 | 8.16E-05 |  |
| cg05313129 | 8 | 58192883 | C8orf71 | 1.24E-04 | 9.66E-02 | -- | -0.06 | -0.06 | -0.06 | 3.72E-05 |  |
| cg22308101 | 8 | 77616048 | ZFHX4 | 1.32E-02 | 1.20E-04 | ++ | 0.02 | 0.05 | 0.03 | 3.01E-05 |  |
| cg09179743 | 9 | 27524436 | MOBKL2B;IFNK | 1.29E-04 | 7.54E-03 | ++ | 0.03 | 0.04 | 0.03 | 3.08E-06 |  |
| cg14364797 | 9 | 132651576 | FNBP1 | 2.65E-04 | 2.45E-02 | ++ | 0.04 | 0.03 | 0.04 | 1.84E-05 |  |
| cg11176472 | 10 | 363662 | DIP2C | 2.05E-04 | 1.37E-02 | ++ | 0.01 | 0.01 | 0.01 | 8.31E-06 |  |
| cg14207539 | 10 | 77794591 | C10orf11 | 5.05E-04 | 4.29E-03 | ++ | 0.01 | 0.02 | 0.02 | 7.74E-06 |  |
| cg26945715 | 10 | 96162066 | TBC1D12 | 6.90E-03 | 2.49E-03 | -- | -0.02 | -0.05 | -0.03 | 9.13E-05 |  |
| cg04149179 | 10 | 97149537 | SORBS1 | 4.58E-03 | 1.37E-03 | ++ | 0.01 | 0.01 | 0.01 | 3.78E-05 |  |
| cg04556008 | 10 | 119293800 | EMX2OS | 9.79E-04 | 2.47E-02 | -- | -0.01 | -0.01 | -0.01 | 6.65E-05 |  |
| cg04915533 | 10 | 120800913 | EIF3A | 8.06E-05 | 2.13E-01 | ++ | 0.03 | 0.01 | 0.02 | 6.75E-05 |  |
| cg10004882 | 10 | 128830453 | DOCK1 | 1.48E-03 | 2.17E-04 | ++ | 0.03 | 0.04 | 0.03 | 2.86E-06 |  |
| cg09512973 | 11 | 1682139 | HCCA2 | 2.71E-02 | 1.76E-04 | ++ | 0.03 | 0.06 | 0.04 | 9.80E-05 |  |
| cg12593634 | 11 | 5143555 | OR52A4 | 4.31E-02 | 2.10E-05 | ++ | 0.02 | 0.04 | 0.03 | 6.01E-05 |  |
| cg16518015 | 11 | 8703871 | RPL27A | 5.38E-04 | 3.12E-02 | -- | -0.01 | -0.01 | -0.01 | 4.57E-05 |  |
| cg09767822 | 11 | 20178040 | DBX1 | 1.14E-02 | 2.02E-04 | -- | -0.04 | -0.06 | -0.05 | 3.40E-05 |  |
| cg13245152 | 11 | 31823238 | PAX6 | 5.29E-03 | 2.20E-03 | -- | -0.03 | -0.07 | -0.04 | 6.19E-05 |  |
| cg20029347 | 11 | 46366877 | DGKZ | 2.55E-02 | 1.08E-04 | -- | -0.03 | -0.06 | -0.04 | 6.83E-05 |  |
| cg10116443 | 11 | 63140804 | SLC22A9 | 2.65E-03 | 1.09E-02 | ++ | 0.02 | 0.02 | 0.02 | 9.33E-05 |  |
| cg00350296 | 11 | 66084841 | CD248 | 4.08E-03 | 5.98E-03 | -- | -0.04 | -0.08 | -0.05 | 9.39E-05 |  |
| cg17196933 | 11 | 121986961 | LOC399959;BLID | 3.30E-04 | 2.12E-02 | ++ | 0.01 | 0.00 | 0.01 | 1.99E-05 |  |
| cg26221631 | 11 | 129245988 | BARX2 | 2.01E-04 | 1.25E-01 | -- | -0.02 | -0.01 | -0.02 | 7.65E-05 |  |
| cg21597754 | 12 | 7310839 | CLSTN3 | 3.83E-03 | 4.84E-03 | ++ | 0.02 | 0.02 | 0.02 | 7.50E-05 |  |
| cg11843304 | 12 | 15501337 | PTPRO | 6.64E-03 | 2.84E-03 | ++ | 0.04 | 0.05 | 0.04 | 9.57E-05 |  |
| cg20985486 | 12 | 56510361 | RPL41 | 1.30E-03 | 1.76E-02 | -- | 0.00 | 0.00 | 0.00 | 6.61E-05 |  |
| cg21913159 | 12 | 57588171 | LRP1;MIR1228 | 2.49E-03 | 4.50E-03 | ++ | 0.02 | 0.03 | 0.03 | 4.44E-05 |  |
| cg24691904 | 12 | 123380870 | VPS37B | 3.32E-03 | 7.95E-04 | -- | -0.03 | -0.04 | -0.03 | 1.79E-05 |  |
| cg02766259 | 12 | 125626809 | AACS | 1.07E-04 | 2.26E-01 | ++ | 0.02 | 0.01 | 0.02 | 9.21E-05 |  |
| cg00674995 | 13 | 42708444 | DGKH | 3.89E-03 | 2.70E-03 | ++ | 0.02 | 0.04 | 0.03 | 5.02E-05 |  |
| cg08029014 | 13 | 52256202 | WDFY2 | 3.36E-02 | 7.54E-05 | ++ | 0.01 | 0.04 | 0.02 | 8.24E-05 |  |
| cg06214253 | 13 | 73438525 | PIBF1 | 2.23E-02 | 4.14E-05 | ++ | 0.02 | 0.03 | 0.02 | 3.35E-05 |  |
| cg09885657 | 13 | 113791997 | F10 | 4.82E-04 | 7.64E-02 | ++ | 0.02 | 0.02 | 0.02 | 9.90E-05 |  |
| cg20019356 | 14 | 64764216 | ESR2 | 1.32E-03 | 2.18E-02 | ++ | 0.03 | 0.02 | 0.03 | 8.03E-05 |  |
| cg17940694 | 15 | 40657813 | DISP2 | 1.96E-03 | 7.26E-04 | -- | -0.02 | -0.03 | -0.02 | 9.13E-06 |  |
| cg01754967 | 15 | 54049269 | WDR72 | 1.27E-03 | 2.35E-02 | ++ | 0.03 | 0.04 | 0.04 | 8.25E-05 |  |
| cg06598982 | 15 | 66437024 | MEGF11 | 1.31E-03 | 2.52E-03 | ++ | 0.01 | 0.01 | 0.01 | 1.43E-05 |  |
| cg01666796 | 15 | 70364327 | TLE3 | 1.95E-03 | 8.52E-03 | ++ | 0.01 | 0.02 | 0.01 | 5.57E-05 |  |
| cg12349571 | 15 | 70364359 | TLE3 | 3.80E-03 | 1.19E-03 | ++ | 0.01 | 0.02 | 0.01 | 2.76E-05 |  |
| cg07217653 | 15 | 101591436 | LRRK1 | 8.47E-02 | 5.54E-06 | ++ | 0.02 | 0.04 | 0.03 | 8.87E-05 |  |
| cg00175153 | 16 | 2012763 | SNORA10;RPS2 | 5.58E-02 | 2.55E-05 | ++ | 0.02 | 0.05 | 0.03 | 9.77E-05 |  |
| cg06139166 | 16 | 67440283 | ZDHHC1 | 9.93E-03 | 1.36E-03 | ++ | 0.01 | 0.01 | 0.01 | 9.38E-05 |  |
| cg04305821 | 16 | 87444933 | ZCCHC14 | 1.41E-02 | 2.30E-04 | ++ | 0.02 | 0.04 | 0.03 | 4.83E-05 |  |
| cg06222638 | 17 | 4542859 | ALOX15 | 2.76E-02 | 8.15E-05 | -- | -0.01 | -0.01 | -0.01 | 6.53E-05 |  |
| cg07540103 | 17 | 6679499 | FBXO39 | 1.05E-02 | 8.19E-04 | -- | -0.02 | -0.03 | -0.02 | 7.24E-05 |  |
| cg26083576 | 17 | 29902249 | MIR365-2 | 6.94E-04 | 4.71E-03 | ++ | 0.01 | 0.01 | 0.01 | 1.17E-05 |  |
| cg12029804 | 17 | 38821390 | KRT222 | 2.66E-03 | 7.41E-03 | -- | -0.04 | -0.04 | -0.04 | 6.94E-05 |  |
| cg18439358 | 17 | 41910968 | MPP3 | 4.45E-04 | 2.40E-02 | -- | -0.01 | -0.01 | -0.01 | 2.98E-05 |  |
| cg25019564 | 18 | 9475492 | RALBP1 | 5.00E-04 | 3.92E-02 | -- | 0.00 | 0.00 | 0.00 | 5.28E-05 |  |
| cg12998614 | 19 | 917068 | KISS1R | 4.32E-03 | 4.34E-05 | -- | -0.03 | -0.04 | -0.03 | 3.84E-06 |  |
| cg27364205 | 19 | 1495210 | REEP6 | 5.12E-05 | 2.57E-01 | ++ | 0.01 | 0.01 | 0.01 | 5.94E-05 |  |
| cg09310112 | 19 | 4969989 | KDM4B | 1.01E-01 | 2.02E-06 | -- | -0.01 | -0.02 | -0.01 | 7.36E-05 |  |
| cg07756483 | 19 | 44281665 | KCNN4 | 4.26E-03 | 2.32E-03 | -- | -0.02 | -0.03 | -0.02 | 4.99E-05 |  |
| cg08844501 | 19 | 51582841 | KLK14 | 3.25E-04 | 9.83E-02 | ++ | 0.02 | 0.01 | 0.01 | 9.03E-05 |  |
| cg19145398 | 20 | 30433673 | FOXS1 | 3.25E-02 | 1.12E-04 | ++ | 0.03 | 0.04 | 0.03 | 9.74E-05 |  |
| cg22407822 | 20 | 57463658 | GNAS | 3.95E-02 | 5.90E-05 | -- | -0.02 | -0.03 | -0.03 | 9.08E-05 |  |
| cg01458054 | 20 | 62200603 | PRIC285 | 1.50E-02 | 6.81E-05 | ++ | 0.03 | 0.06 | 0.04 | 2.57E-05 |  |
| cg12954385 | 21 | 46686990 | POFUT2 | 1.99E-02 | 5.12E-05 | ++ | 0.01 | 0.03 | 0.02 | 3.21E-05 |  |

Only CpGs with P<E-04 that map to a gene body are shown. Direction of beta was consistent in monozygotic (MZ) and dizygotic (DZ) twins. Chr: chromosome, RefGene: reference gene; Dir: direction; W: weighted.

| **Table S2. Most significant canonical pathways, upstream regulators, and diseases and biological functions in differentially methylated genes in whole blood from all twin pairs discordant for SSc.** | |
| --- | --- |
| **Top canonical pathways** | **P-value** |
| Factors promoting cardiogenesis in vertebrates | 2.78E-04 |
| Molecular mechanisms of cancer | 2.90E-04 |
| **Top Upstream regulators** | |
| KLF5 | 6.02E-04 |
| CYP19A1 | 8.64E-04 |
| **Top diseases and biological functions** | |
| **Cancer, Gastrointestinal Disease, Organismal Injury and Abnormalities** | |
| malignant neoplasm of large intestine | 3.79E-11 |
| gastrointestinal tract cancer | 4.42E-10 |
| digestive system cancer | 3.40E-08 |
| breast or colorectal cancer | 1.26E-06 |
| colorectal neoplasia | 1.41E-06 |
| colorectal cancer | 1.49E-06 |
| colon tumor | 2.43E-06 |
| colon cancer | 4.16E-06 |
| **Cancer, Organismal Injury and Abnormalities** |  |
| breast or ovarian cancer | 1.10E-05 |
| adenocarcinoma | 1.78E-05 |
| **Cellular Movement** |  |
| cell movement | 3.39E-05 |
| **Cancer, Gastrointestinal Disease, Organismal Injury and Abnormalities** | |
| gastro-esophageal carcinoma | 4.15E-05 |
| gastroesophageal adenocarcinoma | 4.16E-05 |
| colon carcinoma | 4.77E-05 |
| **Cancer, Organismal Injury and Abnormalities** | |
| abdominal cancer | 4.95E-05 |
| breast or ovarian carcinoma | 6.83E-05 |
| **Cancer, Gastrointestinal Disease, Organismal Injury and Abnormalities** | |
| gastroesophageal cancer | 6.88E-05 |
| colon adenocarcinoma | 8.55E-05 |
| gastrointestinal adenocarcinoma | 8.98E-05 |

| **Table S3. Most significant differentially methylated cytosines in whole blood from twins discordant for lcSSc.** | | | | | | | | | | |
| --- | --- | --- | --- | --- | --- | --- | --- | --- | --- | --- |
| **CpG** | **Chr** | **Position (bp)** | **RefGene** | **MZ P-value** | **DZ P-value** | **Dir** | **MZ Beta** | **DZ Beta** | **W Beta** | **Meta P-value** |
| cg00390654 | 1 | 2214240 | SKI | 6.77E-04 | 8.43E-03 | -- | -0.01 | -0.01 | -0.01 | 2.47E-05 |
| cg01719793 | 1 | 16533434 | ARHGEF19 | 3.34E-04 | 3.68E-02 | -- | -0.01 | 0.00 | 0.00 | 3.44E-05 |
| cg17434062 | 1 | 17312704 | ATP13A2 | 1.45E-04 | 1.42E-01 | -- | -0.03 | -0.05 | -0.04 | 5.00E-05 |
| cg13876650 | 1 | 26146005 | FAM54B | 5.98E-04 | 2.08E-02 | -- | -0.03 | -0.05 | -0.04 | 4.06E-05 |
| cg09269118 | 1 | 51716790 | RNF11 | 2.40E-03 | 5.11E-03 | -- | -0.01 | -0.02 | -0.01 | 7.25E-05 |
| cg13901901 | 1 | 76190435 | ACADM | 5.56E-04 | 4.26E-02 | ++ | 0.00 | 0.00 | 0.00 | 6.49E-05 |
| cg03460527 | 1 | 95008117 | F3 | 3.14E-03 | 1.14E-03 | -- | -0.01 | -0.02 | -0.01 | 4.19E-05 |
| ch.1.2649807R | 1 | 113207215 | CAPZA1 | 1.76E-03 | 1.38E-02 | -- | -0.02 | -0.04 | -0.03 | 9.67E-05 |
| cg14514991 | 1 | 158737820 | OR6N1 | 3.19E-04 | 1.15E-01 | ++ | 0.02 | 0.03 | 0.02 | 8.73E-05 |
| cg14090822 | 1 | 204912731 | NFASC | 5.42E-04 | 1.60E-02 | ++ | 0.01 | 0.00 | 0.01 | 3.02E-05 |
| cg15523958 | 2 | 43864929 | PLEKHH2 | 8.64E-04 | 2.04E-02 | -- | -0.03 | -0.04 | -0.03 | 5.90E-05 |
| cg00793946 | 2 | 46163986 | PRKCE | 1.08E-04 | 1.64E-01 | -- | -0.02 | -0.01 | -0.02 | 4.38E-05 |
| cg08641090 | 2 | 72375141 | CYP26B1 | 9.14E-03 | 3.22E-04 | -- | -0.03 | -0.04 | -0.03 | 8.15E-05 |
| cg00164121 | 2 | 174228024 | CDCA7 | 1.73E-04 | 4.67E-03 | -- | -0.01 | -0.02 | -0.01 | 3.76E-06 |
| cg19742470 | 2 | 182850059 | PPP1R1C | 1.96E-04 | 3.91E-02 | ++ | 0.03 | 0.04 | 0.03 | 2.11E-05 |
| cg18753162 | 2 | 219314121 | VIL1 | 1.27E-03 | 1.25E-02 | -- | -0.01 | -0.01 | -0.01 | 6.34E-05 |
| cg27416337 | 3 | 32748004 | CNOT10 | 5.03E-05 | 3.86E-02 | -- | -0.07 | -0.09 | -0.07 | 5.34E-06 |
| cg18340446 | 3 | 53916291 | ACTR8 | 6.25E-04 | 3.59E-02 | -- | 0.00 | 0.00 | 0.00 | 6.40E-05 |
| cg12193622 | 3 | 101567865 | NFKBIZ | 3.04E-03 | 3.11E-03 | -- | -0.01 | -0.01 | -0.01 | 7.11E-05 |
| cg12965599 | 3 | 147125924 | ZIC1 | 7.25E-05 | 1.75E-02 | -- | -0.05 | -0.06 | -0.05 | 3.99E-06 |
| cg24798995 | 4 | 731576 | PCGF3 | 7.24E-04 | 3.74E-02 | -- | -0.02 | -0.02 | -0.02 | 7.67E-05 |
| cg04976194 | 4 | 26863237 | STIM2 | 7.81E-04 | 4.54E-02 | ++ | 0.01 | 0.01 | 0.01 | 9.65E-05 |
| cg16700392 | 4 | 156680207 | GUCY1B3 | 1.80E-02 | 2.81E-05 | -- | -0.04 | -0.04 | -0.04 | 6.65E-05 |
| cg04476341 | 5 | 669733 | TPPP | 3.03E-04 | 9.46E-02 | -- | -0.02 | -0.05 | -0.03 | 6.92E-05 |
| cg11666982 | 5 | 1293871 | TERT | 6.93E-06 | 4.13E-01 | -- | -0.03 | -0.01 | -0.02 | 1.15E-05 |
| cg26399773 | 5 | 74633655 | HMGCR | 5.13E-04 | 2.22E-02 | ++ | 0.00 | 0.01 | 0.01 | 3.63E-05 |
| cg19972859 | 5 | 139494006 | PURA | 1.63E-03 | 9.72E-03 | -- | -0.01 | -0.03 | -0.02 | 7.05E-05 |
| cg12974258 | 5 | 149493011 | CSF1R | 4.63E-05 | 1.86E-01 | -- | -0.02 | -0.05 | -0.03 | 2.28E-05 |
| cg10608615 | 5 | 169780747 | KCNIP1 | 3.81E-05 | 2.84E-01 | -- | -0.03 | -0.01 | -0.03 | 3.14E-05 |
| cg26615160 | 5 | 174151043 | MSX2 | 5.14E-04 | 1.23E-02 | -- | -0.01 | -0.02 | -0.01 | 2.37E-05 |
| cg24886356 | 5 | 176300261 | UNC5A | 3.96E-04 | 9.84E-02 | -- | -0.04 | -0.04 | -0.04 | 9.31E-05 |
| cg09510246 | 6 | 1684515 | GMDS | 7.15E-05 | 4.32E-01 | -- | -0.01 | -0.02 | -0.02 | 9.49E-05 |
| cg22950391 | 6 | 28109938 | ZNF192 | 2.70E-05 | 6.85E-01 | ++ | 0.00 | 0.00 | 0.00 | 8.30E-05 |
| cg23148731 | 6 | 29697938 | LOC285830 | 1.42E-03 | 1.98E-02 | ++ | 0.01 | 0.02 | 0.01 | 9.79E-05 |
| cg11265836 | 6 | 30074604 | TRIM31 | 5.82E-05 | 7.66E-04 | ++ | 0.02 | 0.03 | 0.02 | 3.39E-07 |
| cg13197078 | 6 | 31963919 | C4B;C4A | 2.62E-05 | 1.70E-01 | -- | -0.01 | -0.01 | -0.01 | 1.22E-05 |
| cg02673305 | 6 | 32066121 | TNXB | 2.00E-05 | 5.06E-01 | -- | -0.01 | -0.01 | -0.01 | 3.92E-05 |
| cg16045677 | 6 | 32362678 | BTNL2 | 1.13E-03 | 1.06E-02 | ++ | 0.01 | 0.02 | 0.01 | 5.00E-05 |
| cg25482983 | 6 | 33135756 | COL11A2 | 1.66E-03 | 1.48E-02 | -- | -0.02 | -0.05 | -0.03 | 9.49E-05 |
| cg15169162 | 6 | 42690308 | PRPH2 | 5.43E-05 | 4.93E-01 | ++ | 0.01 | 0.01 | 0.01 | 8.97E-05 |
| cg00238662 | 6 | 43235803 | TTBK1 | 7.71E-04 | 4.44E-02 | -- | -0.02 | -0.02 | -0.02 | 9.34E-05 |
| cg26777809 | 6 | 53695333 | LRRC1 | 8.91E-05 | 4.58E-02 | ++ | 0.02 | 0.02 | 0.02 | 1.09E-05 |
| cg15416877 | 6 | 108492769 | NR2E1 | 4.23E-03 | 1.14E-04 | -- | -0.03 | -0.06 | -0.04 | 1.83E-05 |
| cg16370720 | 6 | 108907710 | FOXO3 | 4.27E-03 | 1.27E-03 | -- | -0.01 | -0.01 | -0.01 | 6.42E-05 |
| cg23224666 | 6 | 127796287 | C6orf174 | 1.72E-04 | 8.20E-02 | -- | -0.02 | -0.01 | -0.02 | 3.51E-05 |
| cg01832737 | 6 | 166075022 | PDE10A | 2.84E-03 | 1.94E-03 | -- | -0.02 | -0.02 | -0.02 | 5.00E-05 |
| cg09884940 | 7 | 150741161 | ABCB8 | 4.77E-04 | 3.56E-02 | -- | -0.01 | -0.01 | -0.01 | 4.82E-05 |
| cg11823624 | 7 | 150778724 | FASTK;TMUB1 | 6.10E-05 | 1.31E-02 | -- | -0.02 | -0.02 | -0.02 | 2.66E-06 |
| cg18155267 | 8 | 1860385 | ARHGEF10 | 2.76E-04 | 1.37E-01 | -- | -0.03 | -0.02 | -0.03 | 8.91E-05 |
| cg03555325 | 8 | 22458673 | C8orf58 | 4.54E-04 | 6.15E-02 | -- | -0.01 | -0.02 | -0.02 | 7.11E-05 |
| cg14189875 | 8 | 48691176 | PRKDC | 7.98E-06 | 9.15E-01 | ++ | 0.01 | 0.00 | 0.01 | 5.31E-05 |
| cg25456425 | 8 | 143361158 | TSNARE1 | 9.51E-05 | 3.09E-01 | -- | -0.02 | -0.03 | -0.02 | 7.96E-05 |
| cg01115058 | 8 | 145733190 | MFSD3 | 2.26E-04 | 5.14E-02 | -- | -0.04 | -0.04 | -0.04 | 3.05E-05 |
| cg07269146 | 9 | 34710657 | CCL21 | 8.92E-04 | 3.95E-02 | -- | -0.03 | -0.04 | -0.03 | 9.92E-05 |
| cg13639672 | 9 | 139743720 | PHPT1 | 2.52E-04 | 3.34E-02 | ++ | 0.00 | 0.00 | 0.00 | 2.39E-05 |
| cg15106452 | 9 | 140115474 | RNF208 | 4.28E-04 | 4.42E-03 | -- | -0.02 | -0.01 | -0.02 | 9.73E-06 |
| cg18274896 | 9 | 140395754 | PNPLA7 | 5.61E-04 | 3.36E-02 | -- | -0.01 | -0.02 | -0.01 | 5.44E-05 |
| cg20392242 | 10 | 8006677 | TAF3 | 1.34E-03 | 1.93E-04 | -- | -0.01 | -0.01 | -0.01 | 5.73E-06 |
| cg07261186 | 10 | 81070689 | ZMIZ1 | 8.71E-03 | 3.90E-04 | ++ | 0.03 | 0.06 | 0.03 | 8.41E-05 |
| cg05317956 | 10 | 99409824 | PI4K2A | 1.85E-05 | 8.10E-01 | -- | -0.01 | 0.00 | -0.01 | 8.24E-05 |
| cg25937598 | 10 | 105240427 | CALHM3 | 2.49E-04 | 1.41E-01 | -- | -0.03 | -0.03 | -0.03 | 8.36E-05 |
| cg11748187 | 10 | 114713108 | TCF7L2 | 2.69E-03 | 1.33E-03 | -- | -0.06 | -0.10 | -0.07 | 3.79E-05 |
| cg06965072 | 10 | 133956982 | JAKMIP3 | 2.41E-04 | 4.32E-02 | -- | -0.05 | -0.11 | -0.06 | 2.81E-05 |
| cg14353649 | 10 | 135191496 | PAOX | 2.96E-04 | 1.31E-01 | -- | -0.03 | -0.06 | -0.04 | 9.14E-05 |
| cg14585160 | 11 | 46722671 | ZNF408;ARHGAP1 | 4.76E-04 | 7.63E-02 | ++ | 0.01 | 0.01 | 0.01 | 8.93E-05 |
| cg06533679 | 11 | 47374222 | MYBPC3 | 6.18E-05 | 1.34E-01 | -- | -0.03 | -0.02 | -0.02 | 2.10E-05 |
| cg06341513 | 11 | 61277017 | LRRC10B | 1.50E-03 | 7.13E-04 | -- | -0.01 | 0.00 | 0.00 | 1.35E-05 |
| cg18988094 | 11 | 63954227 | STIP1 | 3.16E-04 | 9.97E-02 | ++ | 0.03 | 0.04 | 0.03 | 7.57E-05 |
| cg06378491 | 11 | 64564012 | MAP4K2 | 1.45E-03 | 1.63E-03 | -- | -0.02 | -0.02 | -0.02 | 2.06E-05 |
| cg15056794 | 11 | 121987155 | BLID | 5.51E-04 | 2.52E-02 | ++ | 0.03 | 0.06 | 0.04 | 4.30E-05 |
| cg00292447 | 11 | 126276006 | ST3GAL4 | 6.50E-05 | 1.13E-02 | -- | -0.03 | -0.04 | -0.03 | 2.53E-06 |
| cg01712892 | 11 | 131513454 | NTM | 5.67E-05 | 2.35E-01 | -- | -0.02 | -0.02 | -0.02 | 3.60E-05 |
| cg22804770 | 12 | 2786316 | CACNA1C | 5.15E-04 | 5.72E-02 | -- | -0.01 | -0.03 | -0.02 | 7.61E-05 |
| cg02566627 | 12 | 58232984 | CTDSP2 | 7.10E-04 | 4.84E-03 | -- | -0.03 | -0.04 | -0.03 | 1.80E-05 |
| cg08975197 | 12 | 70173946 | RAB3IP | 6.19E-04 | 4.58E-02 | -- | -0.01 | -0.01 | -0.01 | 7.66E-05 |
| cg11017500 | 13 | 111290755 | CARKD | 3.76E-04 | 3.85E-02 | ++ | 0.02 | 0.02 | 0.02 | 4.01E-05 |
| cg09396850 | 13 | 113400630 | ATP11A | 4.64E-04 | 2.92E-02 | ++ | 0.01 | 0.01 | 0.01 | 4.02E-05 |
| cg02745494 | 13 | 114771098 | RASA3 | 2.10E-04 | 9.15E-02 | -- | -0.02 | 0.00 | -0.02 | 4.69E-05 |
| cg06680369 | 14 | 38679393 | SSTR1 | 1.59E-04 | 2.56E-04 | -- | -0.03 | -0.02 | -0.03 | 5.37E-07 |
| cg13630250 | 14 | 70827336 | COX16 | 1.59E-03 | 2.66E-03 | -- | -0.04 | -0.03 | -0.04 | 3.08E-05 |
| cg25095171 | 14 | 93577304 | ITPK1 | 2.64E-04 | 9.41E-02 | -- | -0.03 | -0.04 | -0.03 | 6.03E-05 |
| cg04184810 | 14 | 101037198 | BEGAIN | 3.28E-03 | 4.16E-03 | -- | -0.02 | -0.04 | -0.02 | 9.18E-05 |
| cg18446701 | 14 | 105357477 | KIAA0284 | 1.73E-03 | 7.09E-04 | -- | -0.01 | -0.02 | -0.02 | 1.59E-05 |
| cg07438412 | 14 | 105852272 | PACS2 | 2.01E-04 | 7.08E-02 | -- | -0.04 | -0.04 | -0.04 | 3.57E-05 |
| cg15532667 | 14 | 105939672 | CRIP2 | 3.43E-04 | 4.46E-02 | -- | -0.04 | -0.05 | -0.04 | 4.12E-05 |
| cg22432269 | 15 | 22892697 | CYFIP1 | 5.86E-05 | 1.65E-02 | -- | -0.01 | -0.01 | -0.01 | 3.06E-06 |
| cg19405229 | 15 | 101593509 | LRRK1 | 7.16E-06 | 4.92E-01 | ++ | 0.01 | 0.00 | 0.00 | 1.54E-05 |
| cg09290694 | 16 | 972115 | LMF1 | 6.74E-04 | 4.11E-02 | ++ | 0.01 | 0.01 | 0.01 | 7.67E-05 |
| cg27018309 | 16 | 8943122 | PMM2 | 5.93E-05 | 3.77E-01 | -- | -0.02 | -0.02 | -0.02 | 6.70E-05 |
| cg08181850 | 16 | 17200244 | XYLT1 | 5.14E-03 | 1.12E-03 | -- | -0.03 | -0.02 | -0.03 | 7.48E-05 |
| cg05470939 | 16 | 56898573 | SLC12A3 | 2.08E-04 | 1.15E-01 | -- | -0.01 | -0.02 | -0.01 | 5.73E-05 |
| cg02236679 | 16 | 78782477 | WWOX | 8.08E-04 | 3.21E-02 | -- | -0.02 | -0.02 | -0.02 | 7.66E-05 |
| cg01657422 | 16 | 79103057 | WWOX | 1.30E-03 | 2.53E-03 | -- | -0.01 | -0.01 | -0.01 | 2.38E-05 |
| cg03092447 | 16 | 89342399 | ANKRD11 | 4.10E-04 | 8.02E-02 | -- | -0.03 | -0.03 | -0.03 | 8.04E-05 |
| cg01739831 | 16 | 89922539 | SPIRE2 | 8.09E-04 | 4.00E-02 | -- | -0.02 | -0.02 | -0.02 | 9.06E-05 |
| cg20216752 | 17 | 6913521 | ALOX12 | 2.88E-04 | 8.81E-02 | -- | -0.05 | -0.08 | -0.05 | 6.17E-05 |
| cg19147912 | 17 | 17596386 | RAI1 | 2.04E-05 | 2.09E-01 | -- | -0.02 | -0.02 | -0.02 | 1.23E-05 |
| cg11646887 | 17 | 45918805 | SCRN2 | 2.58E-04 | 1.02E-01 | -- | -0.01 | -0.01 | -0.01 | 6.34E-05 |
| cg23551720 | 17 | 46633726 | HOXB3 | 6.99E-05 | 3.34E-01 | -- | -0.02 | -0.01 | -0.02 | 6.65E-05 |
| cg18032289 | 17 | 61959525 | GH2 | 5.48E-05 | 3.29E-01 | -- | -0.03 | -0.02 | -0.03 | 5.23E-05 |
| cg21678736 | 17 | 75282776 | SEPT9 | 7.02E-03 | 8.09E-04 | -- | -0.02 | -0.03 | -0.02 | 9.26E-05 |
| cg24682036 | 18 | 29077698 | DSG2 | 2.24E-03 | 5.15E-03 | -- | -0.03 | -0.06 | -0.04 | 6.75E-05 |
| cg08857677 | 19 | 5341595 | PTPRS | 2.79E-04 | 7.58E-02 | -- | -0.02 | -0.04 | -0.02 | 5.25E-05 |
| cg18592083 | 19 | 9731957 | ZNF561 | 5.44E-04 | 4.52E-02 | ++ | 0.00 | 0.00 | 0.00 | 6.64E-05 |
| cg06716436 | 19 | 10342240 | S1PR2 | 2.46E-04 | 1.40E-02 | -- | -0.01 | -0.01 | -0.01 | 1.19E-05 |
| cg12087891 | 19 | 11304498 | KANK2 | 1.65E-04 | 2.55E-02 | -- | -0.02 | -0.01 | -0.02 | 1.25E-05 |
| cg23994702 | 19 | 12833615 | TNPO2 | 3.21E-04 | 1.00E-01 | -- | -0.01 | -0.01 | -0.01 | 7.70E-05 |
| cg03202884 | 19 | 19248891 | TMEM161A | 1.39E-03 | 1.41E-02 | -- | -0.04 | -0.04 | -0.04 | 7.60E-05 |
| cg27181142 | 19 | 46270322 | SIX5 | 1.61E-04 | 2.33E-01 | -- | -0.02 | -0.01 | -0.02 | 9.28E-05 |
| cg13099813 | 19 | 50292059 | AP2A1 | 7.56E-03 | 3.00E-04 | -- | -0.02 | -0.02 | -0.02 | 6.17E-05 |
| cg23961059 | 19 | 57874869 | ZNF547;TRAPPC2P1 | 9.51E-05 | 2.69E-01 | ++ | 0.00 | 0.01 | 0.00 | 6.74E-05 |
| cg20618996 | 20 | 33562784 | MYH7B | 8.84E-05 | 1.86E-02 | -- | -0.01 | -0.02 | -0.01 | 5.15E-06 |
| cg26534489 | 20 | 57427495 | GNAS | 2.55E-03 | 8.25E-04 | -- | -0.02 | -0.04 | -0.03 | 2.74E-05 |
| cg24074477 | 22 | 17956455 | CECR2 | 3.01E-04 | 4.93E-02 | -- | -0.07 | -0.06 | -0.07 | 3.93E-05 |
| cg11138192 | 22 | 39965842 | CACNA1I | 1.95E-04 | 1.90E-01 | ++ | 0.02 | 0.02 | 0.02 | 8.89E-05 |
| cg05602642 | 22 | 47081634 | CERK | 3.94E-04 | 5.53E-02 | -- | -0.04 | -0.05 | -0.04 | 5.65E-05 |

Only CpGs with P<E-04 that map to a gene body are shown. Direction of beta was consistent in monozygotic (MZ) and dizygotic (DZ) twins. Chr: chromosome, RefGene: reference gene; Dir: direction; W: weighted.

| **Table S4. Most significant canonical pathways, upstream regulators, and diseases and biological functions in differentially methylated genes in whole blood from twin pairs discordant for lcSSc.** | |
| --- | --- |
| **Top canonical pathways** | **P-value** |
| GABA receptor signaling | 4.64E-03 |
| **Top Upstream regulators** |  |
| SP3 | 1.92E-04 |
| PKNOX1 | 5.43E-04 |
| SP1 | 6.05E-04 |
| **Top diseases and biological functions** |  |
| **Cancer, Endocrine System Disorders, Gastrointestinal Disease, Organismal Injury and Abnormalities** | |
| pancreatic tumor | 1.80E-07 |
| carcinoma in pancreas | 2.18E-07 |
| pancreatic cancer | 2.94E-07 |
| cancer of secretory structure | 2.95E-07 |
| malignant neoplasm of large intestine | 1.79E-06 |
| ductal pancreatic carcinoma | 2.78E-06 |
| adenocarcinoma | 4.57E-06 |
| gastrointestinal tract cancer | 2.53E-05 |
| **Cardiovascular Disease** |  |
| coronary disease | 2.63E-05 |
| atherosclerosis | 3.75E-05 |
| **Cancer, Gastrointestinal Disease, Organismal Injury and Abnormalities** | |
| digestive organ tumor | 4.84E-05 |
| digestive system cancer | 5.73E-05 |
| **Renal and Urological Disease** |  |
| renal impairment | 7.37E-05 |
| **Cell-to-Cell Signaling and Interaction** |  |
| activation of lung cancer cell lines | 8.42E-05 |
| **Cardiovascular Disease** |  |
| primary hypertension | 8.48E-05 |
| **Cell Death and Survival** |  |
| delay in cell death | 9.55E-05 |
| **Cardiovascular Disease, Hereditary Disorder, Organismal Injury and Abnormalities, Skeletal and Muscular Disorders** | |
| Brugada syndrome type 1 | 1.19E-04 |
| **Cardiovascular Disease, Organismal Injury and Abnormalities** | |
| coronary artery disease | 1.34E-04 |
| **Cancer, Organismal Injury and Abnormalities** |  |
| abdominal neoplasm | 1.51E-04 |
| abdominal cancer | 1.63E-04 |
| **Cellular Assembly and Organization** |  |
| formation of cytoskeleton | 1.74E-04 |
| development of cytoplasm | 2.49E-04 |
| formation of filaments | 2.67E-04 |
| **Connective Tissue Disorders, Inflammatory Diseases, Skeletal and Muscular Disorders** | |
| inflammation of joint | 2.70E-04 |
| **Cancer, Endocrine System Disorders, Organismal Injury and Abnormalities** | |
| secreting adrenocortical adenoma | 2.78E-04 |
| **Cancer, Organismal Injury and Abnormalities** |  |
| tumorigenesis of tissues | 2.88E-04 |
| **Cell Morphology** |  |
| modification of gonadal cell lines | 3.09E-04 |
| **Cardiovascular Disease, Organismal Injury and Abnormalities** | |
| long QT-syndrome | 3.42E-04 |
| **Cell Cycle** |  |
| arrest in interphase of pancreatic cancer cell lines | 3.51E-04 |
| **Cardiovascular Disease, Developmental Disorder, Hereditary Disorder, Organismal Injury and Abnormalities** | |
| congenital long QT syndrome | 4.36E-04 |

| **Table S5. Most significant differentially methylated cytosines in whole blood from twins discordant for dcSSc.** | | | | | | | | | | |
| --- | --- | --- | --- | --- | --- | --- | --- | --- | --- | --- |
| **CpG** | **Chr** | **Position (bp)** | **RefGene** | **MZ P-value** | **DZ P-value** | **Dir** | **MZ Beta** | **DZ Beta** | **W Beta** | **Meta P-value** |
| cg06219660 | 1 | 1695509 | NADK | 2.37E-05 | 2.16E-01 | -- | -0.10 | -0.21 | -0.14 | 3.10E-05 |
| cg15392054 | 1 | 2271665 | MORN1 | 1.40E-03 | 2.53E-02 | ++ | 0.05 | 0.09 | 0.07 | 9.65E-05 |
| cg08160128 | 1 | 3158200 | PRDM16 | 3.01E-04 | 6.53E-02 | -- | -0.02 | 0.00 | -0.01 | 5.93E-05 |
| cg05599723 | 1 | 12241073 | TNFRSF1B | 5.71E-05 | 2.55E-01 | -- | -0.10 | -0.21 | -0.13 | 8.03E-05 |
| cg06365898 | 1 | 16533907 | ARHGEF19 | 9.59E-03 | 2.28E-04 | -- | -0.03 | -0.12 | -0.06 | 2.21E-05 |
| cg00932104 | 1 | 19972707 | NBL1 | 5.14E-05 | 2.72E-01 | -- | -0.06 | -0.09 | -0.07 | 8.11E-05 |
| cg24585377 | 1 | 26857774 | RPS6KA1 | 2.30E-05 | 1.92E-01 | -- | -0.08 | -0.13 | -0.10 | 2.56E-05 |
| cg24534743 | 1 | 27884345 | AHDC1 | 5.29E-07 | 1.78E-01 | -- | -0.08 | -0.15 | -0.10 | 1.10E-06 |
| cg11226328 | 1 | 29213367 | EPB41 | 7.74E-03 | 2.27E-03 | -- | 0.00 | -0.01 | 0.00 | 8.27E-05 |
| cg23202722 | 1 | 33793808 | PHC2 | 7.94E-05 | 1.85E-01 | -- | -0.11 | -0.22 | -0.15 | 6.67E-05 |
| cg16348470 | 1 | 34642609 | C1orf94 | 4.80E-03 | 4.18E-03 | -- | -0.05 | -0.05 | -0.05 | 7.62E-05 |
| cg26331625 | 1 | 42128098 | HIVEP3 | 1.18E-02 | 1.17E-03 | -- | -0.01 | 0.00 | -0.01 | 8.48E-05 |
| cg25851425 | 1 | 53019484 | ZCCHC11 | 3.14E-05 | 2.89E-01 | -- | -0.01 | 0.00 | -0.01 | 6.05E-05 |
| cg18854765 | 1 | 74665362 | LRRIQ3;TNNI3K;FPGT | 3.75E-05 | 2.62E-01 | -- | -0.09 | -0.16 | -0.12 | 5.98E-05 |
| cg21546950 | 1 | 77904032 | AK5 | 1.34E-04 | 1.10E-02 | ++ | 0.04 | 0.03 | 0.04 | 4.53E-06 |
| cg03607951 | 1 | 79085586 | IFI44L | 1.97E-04 | 8.66E-02 | -- | -0.16 | -0.18 | -0.17 | 5.60E-05 |
| cg08361238 | 1 | 117452304 | PTGFRN | 2.66E-03 | 4.95E-03 | -- | -0.03 | -0.05 | -0.03 | 4.58E-05 |
| cg04127455 | 1 | 156572731 | GPATCH4 | 3.32E-06 | 1.30E-01 | -- | -0.05 | -0.08 | -0.06 | 3.01E-06 |
| cg26104196 | 1 | 159914191 | IGSF9 | 1.42E-04 | 1.69E-01 | -- | -0.04 | -0.07 | -0.05 | 9.61E-05 |
| cg08864944 | 1 | 164633485 | PBX1 | 8.37E-06 | 2.74E-01 | -- | -0.09 | -0.15 | -0.11 | 1.96E-05 |
| cg01910727 | 1 | 226842455 | ITPKB | 8.93E-05 | 1.46E-01 | -- | -0.06 | -0.13 | -0.08 | 5.37E-05 |
| cg08352575 | 2 | 10916238 | ATP6V1C2 | 8.98E-03 | 7.41E-04 | -- | -0.02 | -0.03 | -0.02 | 4.48E-05 |
| cg02069619 | 2 | 11885266 | LPIN1 | 1.77E-04 | 1.22E-01 | -- | -0.07 | -0.08 | -0.07 | 7.70E-05 |
| cg05057534 | 2 | 28497669 | BRE | 8.50E-05 | 2.27E-01 | -- | -0.13 | -0.21 | -0.16 | 9.38E-05 |
| cg24738611 | 2 | 29149895 | WDR43;SNORD53 | 2.01E-05 | 2.37E-01 | -- | -0.10 | -0.15 | -0.11 | 3.12E-05 |
| cg13652008 | 2 | 33359356 | LTBP1 | 3.76E-05 | 2.24E-01 | -- | -0.09 | -0.22 | -0.14 | 4.74E-05 |
| cg00841141 | 2 | 37416819 | SULT6B1 | 5.81E-05 | 2.36E-01 | -- | -0.13 | -0.18 | -0.15 | 7.27E-05 |
| cg14480116 | 2 | 65594890 | SPRED2 | 1.52E-05 | 2.57E-01 | -- | -0.14 | -0.21 | -0.16 | 2.84E-05 |
| cg23527387 | 2 | 100056660 | REV1 | 1.13E-02 | 1.14E-03 | -- | -0.04 | -0.07 | -0.05 | 7.91E-05 |
| cg14131038 | 2 | 106360709 | NCK2 | 7.96E-05 | 1.52E-01 | -- | -0.09 | -0.21 | -0.13 | 5.13E-05 |
| cg02718078 | 2 | 131716968 | ARHGEF4 | 2.35E-04 | 8.06E-02 | ++ | 0.02 | 0.02 | 0.02 | 6.02E-05 |
| cg22659356 | 2 | 169104591 | STK39 | 2.04E-04 | 3.17E-02 | -- | -0.01 | -0.02 | -0.01 | 1.94E-05 |
| cg01462353 | 2 | 169939873 | DHRS9 | 5.49E-05 | 2.59E-01 | -- | -0.11 | -0.20 | -0.14 | 7.95E-05 |
| cg06547715 | 2 | 218990976 | CXCR2 | 2.94E-05 | 2.41E-01 | -- | -0.07 | -0.16 | -0.10 | 4.35E-05 |
| cg08098128 | 2 | 220112465 | STK16 | 1.28E-03 | 1.57E-02 | -- | -0.01 | -0.01 | -0.01 | 5.73E-05 |
| cg04730665 | 2 | 236403704 | AGAP1 | 1.47E-05 | 2.41E-01 | -- | -0.03 | -0.03 | -0.03 | 2.49E-05 |
| cg05958206 | 2 | 236784116 | AGAP1 | 6.82E-03 | 2.48E-03 | ++ | 0.07 | 0.10 | 0.08 | 7.63E-05 |
| cg14414903 | 2 | 240171712 | HDAC4 | 5.47E-05 | 2.63E-01 | -- | -0.09 | -0.13 | -0.11 | 8.12E-05 |
| cg16967583 | 2 | 241807859 | AGXT | 1.10E-04 | 2.03E-01 | -- | -0.09 | -0.15 | -0.11 | 9.89E-05 |
| cg10317164 | 3 | 10274142 | IRAK2 | 2.95E-05 | 2.43E-01 | -- | -0.10 | -0.15 | -0.12 | 4.42E-05 |
| cg03378003 | 3 | 47058276 | SETD2 | 2.78E-03 | 6.47E-03 | -- | -0.01 | -0.02 | -0.02 | 5.97E-05 |
| cg26862175 | 3 | 52528955 | STAB1 | 1.50E-05 | 1.92E-01 | -- | -0.04 | -0.13 | -0.07 | 1.80E-05 |
| cg09447811 | 3 | 121972621 | CASR | 7.30E-05 | 2.29E-01 | -- | -0.10 | -0.15 | -0.11 | 8.40E-05 |
| cg00218914 | 3 | 129146731 | C3orf25 | 8.84E-05 | 1.82E-01 | -- | -0.08 | -0.12 | -0.09 | 7.16E-05 |
| cg07753510 | 3 | 171111243 | TNIK | 3.80E-05 | 1.14E-01 | -- | -0.07 | -0.07 | -0.07 | 1.90E-05 |
| cg02371119 | 3 | 171527346 | PLD1 | 3.06E-03 | 9.27E-03 | -- | -0.02 | -0.03 | -0.02 | 8.85E-05 |
| cg21831174 | 3 | 187011187 | MASP1 | 4.26E-05 | 3.15E-01 | ++ | 0.06 | 0.04 | 0.05 | 8.79E-05 |
| cg05685023 | 3 | 187870940 | LPP | 6.32E-06 | 4.97E-01 | -- | -0.04 | -0.05 | -0.04 | 4.52E-05 |
| cg13346655 | 3 | 188951264 | TPRG1 | 3.26E-03 | 5.91E-03 | -- | -0.03 | -0.04 | -0.03 | 6.58E-05 |
| cg07258167 | 4 | 17514207 | QDPR | 6.80E-06 | 1.61E-01 | -- | -0.04 | -0.06 | -0.04 | 7.37E-06 |
| cg04747180 | 4 | 39116218 | KLHL5 | 4.32E-05 | 3.33E-01 | -- | -0.12 | -0.16 | -0.14 | 9.68E-05 |
| cg02394698 | 4 | 86594376 | ARHGAP24 | 8.57E-05 | 1.86E-01 | -- | -0.12 | -0.17 | -0.14 | 7.14E-05 |
| cg13324779 | 4 | 86923558 | ARHGAP24 | 1.49E-04 | 9.40E-02 | ++ | 0.01 | 0.02 | 0.01 | 4.84E-05 |
| cg05573550 | 4 | 148890890 | ARHGAP10 | 1.26E-04 | 1.56E-01 | -- | -0.08 | -0.11 | -0.09 | 7.81E-05 |
| cg13282486 | 4 | 183814928 | DCTD | 4.29E-04 | 4.49E-02 | ++ | 0.01 | 0.01 | 0.01 | 5.49E-05 |
| cg08282812 | 5 | 1505402 | LPCAT1 | 7.47E-05 | 1.28E-01 | -- | -0.07 | -0.13 | -0.09 | 3.91E-05 |
| cg14518948 | 5 | 68484768 | CENPH | 5.08E-03 | 4.71E-04 | -- | -0.03 | -0.06 | -0.04 | 1.66E-05 |
| cg26269677 | 5 | 76251688 | CRHBP | 1.28E-04 | 4.90E-02 | -- | -0.08 | -0.15 | -0.10 | 2.00E-05 |
| cg16703762 | 5 | 76932047 | OTP | 2.03E-03 | 5.59E-04 | -- | -0.05 | -0.06 | -0.05 | 6.41E-06 |
| cg24866363 | 5 | 80050837 | MSH3 | 6.90E-06 | 8.49E-02 | -- | -0.01 | -0.04 | -0.02 | 3.07E-06 |
| cg05936516 | 5 | 114507066 | TRIM36 | 5.48E-04 | 1.22E-02 | -- | -0.05 | -0.09 | -0.06 | 1.96E-05 |
| cg19696103 | 5 | 132354130 | ZCCHC10 | 5.64E-05 | 2.51E-01 | -- | -0.12 | -0.13 | -0.12 | 7.80E-05 |
| cg14724918 | 5 | 133984172 | SEC24A | 2.38E-03 | 9.01E-03 | -- | -0.01 | -0.01 | -0.01 | 6.64E-05 |
| cg24628744 | 5 | 134735654 | H2AFY | 2.36E-04 | 4.11E-02 | -- | -0.06 | -0.15 | -0.09 | 2.89E-05 |
| cg11670060 | 5 | 145316416 | SH3RF2 | 1.93E-04 | 1.80E-02 | -- | -0.05 | -0.03 | -0.04 | 1.03E-05 |
| cg01152073 | 5 | 179246575 | SQSTM1 | 1.15E-04 | 1.83E-01 | -- | -0.06 | -0.11 | -0.07 | 8.94E-05 |
| cg10568066 | 6 | 30039442 | RNF39 | 1.24E-02 | 1.22E-03 | -- | -0.10 | -0.12 | -0.11 | 9.27E-05 |
| cg17080697 | 6 | 30297382 | TRIM39 | 5.93E-05 | 2.25E-01 | -- | -0.08 | -0.15 | -0.10 | 6.89E-05 |
| cg09281154 | 6 | 30852777 | DDR1 | 5.09E-05 | 4.56E-02 | ++ | 0.00 | 0.01 | 0.00 | 8.12E-06 |
| cg04276715 | 6 | 33254460 | WDR46 | 5.11E-05 | 5.19E-02 | -- | -0.04 | -0.10 | -0.06 | 9.45E-06 |
| cg01679682 | 6 | 37427761 | FTSJD2 | 1.08E-03 | 3.96E-04 | -- | -0.02 | -0.03 | -0.02 | 2.41E-06 |
| cg08053935 | 6 | 41691270 | TFEB | 2.21E-05 | 2.42E-01 | -- | -0.08 | -0.15 | -0.10 | 3.48E-05 |
| cg24815792 | 6 | 42927959 | GNMT | 2.12E-04 | 8.59E-02 | -- | -0.08 | -0.13 | -0.09 | 5.92E-05 |
| cg23004174 | 6 | 74404879 | CD109 | 1.01E-04 | 1.17E-01 | -- | -0.03 | -0.03 | -0.03 | 4.51E-05 |
| cg26684776 | 6 | 90085550 | RRAGD | 1.28E-03 | 1.57E-03 | -- | -0.01 | -0.01 | -0.01 | 8.38E-06 |
| cg16823105 | 6 | 97372572 | KLHL32 | 5.66E-05 | 2.09E-01 | -- | 0.00 | 0.00 | 0.00 | 5.99E-05 |
| cg18924324 | 6 | 100057143 | PRDM13 | 8.45E-03 | 1.41E-03 | -- | -0.03 | -0.04 | -0.03 | 6.49E-05 |
| cg05230906 | 6 | 107391505 | BEND3 | 1.31E-02 | 8.60E-04 | ++ | 0.01 | 0.01 | 0.01 | 7.84E-05 |
| cg15401642 | 6 | 107832670 | SOBP | 1.62E-04 | 7.72E-02 | ++ | 0.02 | 0.03 | 0.03 | 4.14E-05 |
| cg03120555 | 7 | 630473 | PRKAR1B | 5.16E-05 | 1.60E-01 | -- | -0.13 | -0.13 | -0.13 | 3.85E-05 |
| cg25422992 | 7 | 2317059 | SNX8 | 1.04E-02 | 1.15E-03 | ++ | 0.02 | 0.00 | 0.02 | 7.21E-05 |
| cg11884933 | 7 | 2774414 | GNA12 | 2.85E-05 | 2.14E-01 | -- | -0.11 | -0.17 | -0.13 | 3.55E-05 |
| cg07302959 | 7 | 92198639 | FAM133B | 8.67E-05 | 1.95E-01 | -- | -0.10 | -0.18 | -0.13 | 7.74E-05 |
| cg25664034 | 7 | 128469891 | FLNC | 1.21E-04 | 3.00E-02 | -- | -0.05 | -0.05 | -0.05 | 1.12E-05 |
| cg05904013 | 7 | 128579933 | IRF5 | 8.58E-05 | 2.31E-01 | -- | -0.09 | -0.16 | -0.11 | 9.67E-05 |
| cg01475577 | 7 | 128829615 | SMO | 2.83E-04 | 1.01E-01 | -- | -0.04 | -0.08 | -0.05 | 9.17E-05 |
| cg12978800 | 7 | 151403164 | PRKAG2 | 3.87E-04 | 8.35E-02 | -- | -0.04 | -0.09 | -0.06 | 9.73E-05 |
| cg19509988 | 7 | 157460657 | PTPRN2 | 1.23E-04 | 6.84E-02 | ++ | 0.03 | 0.02 | 0.02 | 2.82E-05 |
| cg26470958 | 8 | 6666420 | XKR5 | 2.91E-04 | 6.37E-02 | ++ | 0.03 | 0.01 | 0.03 | 5.60E-05 |
| cg26148020 | 8 | 13136639 | DLC1 | 5.04E-04 | 3.49E-02 | ++ | 0.03 | 0.01 | 0.03 | 4.94E-05 |
| cg03399239 | 8 | 21952741 | FAM160B2 | 2.27E-05 | 3.05E-01 | -- | -0.05 | -0.07 | -0.06 | 5.10E-05 |
| cg21320567 | 8 | 67975880 | CSPP1;COPS5 | 1.67E-04 | 1.46E-01 | -- | -0.12 | -0.24 | -0.16 | 9.15E-05 |
| cg23677272 | 8 | 70748063 | SLCO5A1 | 1.68E-05 | 2.44E-01 | -- | -0.07 | -0.10 | -0.08 | 2.84E-05 |
| cg13335567 | 8 | 71127379 | NCOA2 | 2.41E-03 | 8.11E-03 | -- | -0.08 | -0.03 | -0.06 | 6.16E-05 |
| cg00219816 | 8 | 96280555 | C8orf37 | 2.40E-05 | 1.67E-01 | -- | -0.05 | -0.11 | -0.07 | 2.16E-05 |
| cg08598221 | 8 | 121824929 | SNTB1 | 5.55E-06 | 3.45E-01 | -- | -0.08 | -0.11 | -0.09 | 2.10E-05 |
| cg13630493 | 9 | 36190154 | CLTA | 7.60E-03 | 5.61E-04 | -- | -0.08 | -0.10 | -0.08 | 3.03E-05 |
| cg00927495 | 9 | 96715687 | BARX1 | 7.68E-05 | 3.63E-02 | -- | -0.01 | -0.01 | -0.01 | 9.12E-06 |
| cg12044689 | 9 | 97203357 | HIATL1 | 1.58E-03 | 5.79E-03 | -- | -0.02 | -0.01 | -0.02 | 3.00E-05 |
| cg01295399 | 10 | 50820278 | SLC18A3;CHAT | 3.03E-04 | 4.80E-02 | -- | -0.04 | -0.03 | -0.04 | 4.28E-05 |
| cg20418394 | 10 | 72254335 | KIAA1274 | 7.52E-04 | 1.45E-03 | -- | -0.05 | -0.09 | -0.06 | 4.44E-06 |
| cg14207539 | 10 | 77794591 | C10orf11 | 7.79E-04 | 4.43E-03 | ++ | 0.03 | 0.01 | 0.02 | 1.15E-05 |
| cg25824127 | 10 | 115926060 | C10orf118 | 3.95E-03 | 6.14E-03 | -- | -0.02 | -0.02 | -0.02 | 8.32E-05 |
| cg10206933 | 10 | 133786713 | BNIP3 | 8.67E-05 | 2.06E-01 | ++ | 0.09 | 0.08 | 0.09 | 8.34E-05 |
| cg23687466 | 11 | 504937 | RNH1 | 3.75E-05 | 2.72E-01 | -- | -0.11 | -0.09 | -0.10 | 6.34E-05 |
| cg18313182 | 11 | 818903 | PNPLA2 | 1.16E-05 | 1.45E-01 | -- | -0.09 | -0.18 | -0.12 | 9.75E-06 |
| cg03450829 | 11 | 1772468 | HCCA2 | 2.92E-05 | 2.43E-01 | -- | -0.05 | -0.08 | -0.06 | 4.37E-05 |
| cg14020320 | 11 | 2323851 | TSPAN32;C11orf21 | 2.25E-03 | 2.01E-03 | -- | -0.02 | -0.03 | -0.02 | 1.88E-05 |
| cg07824422 | 11 | 2555406 | KCNQ1 | 2.66E-05 | 2.70E-01 | -- | -0.07 | -0.11 | -0.08 | 4.78E-05 |
| cg21019522 | 11 | 2920789 | SLC22A18 | 4.72E-05 | 2.86E-01 | -- | -0.07 | -0.12 | -0.09 | 8.18E-05 |
| cg06005892 | 11 | 3177622 | OSBPL5 | 5.28E-05 | 1.53E-01 | -- | -0.09 | -0.14 | -0.11 | 3.68E-05 |
| cg05645677 | 11 | 20621109 | SLC6A5 | 3.18E-03 | 9.00E-03 | -- | -0.06 | -0.13 | -0.08 | 8.98E-05 |
| cg20360688 | 11 | 26544953 | ANO3 | 9.73E-04 | 3.58E-02 | -- | -0.02 | -0.01 | -0.02 | 9.43E-05 |
| cg08939373 | 11 | 33563246 | C11orf41 | 1.41E-04 | 1.51E-01 | -- | -0.07 | -0.21 | -0.12 | 8.23E-05 |
| cg11925488 | 11 | 45825579 | SLC35C1 | 1.83E-05 | 3.82E-01 | -- | -0.04 | -0.05 | -0.04 | 6.23E-05 |
| cg17330838 | 11 | 57267101 | SLC43A1 | 2.79E-05 | 3.53E-01 | -- | -0.03 | -0.04 | -0.04 | 7.57E-05 |
| cg11479156 | 11 | 70672388 | SHANK2 | 2.66E-04 | 6.10E-02 | -- | -0.03 | -0.05 | -0.04 | 4.93E-05 |
| cg13912027 | 11 | 72759293 | FCHSD2 | 3.16E-04 | 8.44E-02 | -- | -0.05 | -0.05 | -0.05 | 8.23E-05 |
| cg04099543 | 11 | 72983405 | P2RY6 | 3.08E-05 | 2.70E-01 | -- | -0.06 | -0.14 | -0.09 | 5.35E-05 |
| cg09416908 | 11 | 86384670 | ME3 | 6.05E-05 | 2.55E-01 | -- | -0.12 | -0.18 | -0.14 | 8.42E-05 |
| cg20073686 | 11 | 105481863 | GRIA4 | 7.45E-03 | 1.29E-04 | -- | -0.02 | -0.03 | -0.02 | 1.11E-05 |
| cg01142676 | 11 | 117695591 | FXYD2 | 3.83E-05 | 2.15E-01 | -- | -0.10 | -0.14 | -0.11 | 4.56E-05 |
| cg01692842 | 11 | 134126364 | ACAD8 | 1.23E-05 | 1.74E-01 | -- | -0.11 | -0.25 | -0.15 | 1.33E-05 |
| cg19069360 | 12 | 1922058 | CACNA2D4 | 4.54E-05 | 2.60E-01 | -- | -0.11 | -0.18 | -0.13 | 6.90E-05 |
| cg26254667 | 12 | 6452518 | TNFRSF1A | 8.51E-06 | 4.98E-01 | ++ | 0.03 | 0.01 | 0.02 | 5.66E-05 |
| cg07052231 | 12 | 7363540 | PEX5 | 1.93E-05 | 2.54E-01 | -- | -0.10 | -0.17 | -0.12 | 3.36E-05 |
| cg06611426 | 12 | 52404161 | GRASP | 3.41E-05 | 1.79E-01 | -- | -0.07 | -0.12 | -0.08 | 3.19E-05 |
| cg09802818 | 12 | 52604609 | LOC283404 | 1.26E-05 | 2.44E-01 | -- | -0.09 | -0.15 | -0.11 | 2.25E-05 |
| cg10118167 | 12 | 94676545 | PLXNC1 | 6.17E-05 | 2.04E-01 | -- | -0.05 | -0.05 | -0.05 | 6.21E-05 |
| cg07905054 | 12 | 102272136 | DRAM1 | 9.45E-05 | 1.82E-01 | -- | -0.07 | -0.09 | -0.08 | 7.53E-05 |
| cg01686739 | 12 | 107855547 | BTBD11 | 1.77E-05 | 3.45E-01 | -- | -0.06 | -0.08 | -0.07 | 5.13E-05 |
| cg08865208 | 12 | 118469768 | RFC5 | 8.00E-03 | 8.76E-04 | -- | 0.00 | -0.01 | -0.01 | 4.38E-05 |
| cg04347477 | 12 | 125002007 | NCOR2 | 3.32E-06 | 2.70E-01 | -- | -0.09 | -0.10 | -0.10 | 9.26E-06 |
| cg17187521 | 12 | 125003379 | NCOR2 | 4.63E-05 | 2.16E-01 | -- | -0.11 | -0.14 | -0.12 | 5.33E-05 |
| cg26776551 | 13 | 51944507 | INTS6 | 3.23E-05 | 2.44E-01 | -- | -0.11 | -0.18 | -0.13 | 4.78E-05 |
| cg24062389 | 13 | 103478447 | BIVM | 9.58E-03 | 4.89E-04 | -- | -0.04 | -0.06 | -0.04 | 3.65E-05 |
| cg22535089 | 13 | 114184462 | TMCO3 | 5.25E-05 | 2.06E-01 | -- | -0.13 | -0.17 | -0.14 | 5.54E-05 |
| cg17315426 | 14 | 23527629 | CDH24 | 9.18E-05 | 1.83E-01 | -- | -0.03 | -0.06 | -0.04 | 7.42E-05 |
| cg05142272 | 14 | 24896538 | CBLN3 | 1.34E-04 | 1.63E-01 | -- | -0.07 | -0.14 | -0.09 | 8.69E-05 |
| cg11036936 | 14 | 68285471 | RAD51L1 | 1.77E-04 | 1.37E-01 | -- | -0.07 | -0.12 | -0.09 | 8.84E-05 |
| cg21875274 | 14 | 74226445 | C14orf43 | 3.31E-03 | 4.22E-03 | -- | -0.01 | -0.01 | -0.01 | 5.11E-05 |
| cg02268192 | 14 | 92981666 | RIN3 | 1.17E-05 | 2.59E-01 | -- | -0.09 | -0.12 | -0.10 | 2.33E-05 |
| cg01626885 | 15 | 45937757 | SQRDL | 4.91E-05 | 2.30E-01 | -- | -0.09 | -0.14 | -0.10 | 6.13E-05 |
| cg17232357 | 15 | 67012832 | SMAD6 | 1.18E-03 | 2.69E-02 | -- | -0.05 | -0.09 | -0.06 | 8.61E-05 |
| cg16223079 | 15 | 75339541 | PPCDC | 2.59E-06 | 3.32E-01 | -- | -0.04 | -0.07 | -0.05 | 1.09E-05 |
| cg09784977 | 15 | 83361057 | AP3B2 | 6.52E-04 | 6.09E-03 | -- | -0.01 | 0.00 | -0.01 | 1.26E-05 |
| cg16558770 | 15 | 90548037 | ZNF710 | 1.03E-04 | 1.62E-01 | -- | -0.13 | -0.20 | -0.15 | 6.96E-05 |
| cg00005390 | 16 | 2569281 | AMDHD2;ATP6V0C | 2.23E-04 | 8.13E-02 | -- | -0.04 | -0.01 | -0.03 | 5.80E-05 |
| cg01662869 | 16 | 4730410 | MGRN1 | 3.30E-05 | 2.54E-01 | -- | -0.10 | -0.17 | -0.12 | 5.16E-05 |
| cg00616572 | 16 | 10772249 | TEKT5 | 3.98E-05 | 5.29E-02 | ++ | 0.03 | 0.02 | 0.03 | 7.74E-06 |
| cg03308839 | 16 | 15797297 | NDE1;MYH11 | 4.65E-05 | 2.72E-01 | -- | -0.11 | -0.22 | -0.15 | 7.53E-05 |
| cg04045089 | 16 | 21170107 | DNAH3;TMEM159 | 3.68E-04 | 5.92E-02 | -- | -0.01 | -0.01 | -0.01 | 6.40E-05 |
| cg08961793 | 16 | 28628118 | SULT1A1 | 1.15E-04 | 1.74E-01 | -- | -0.12 | -0.16 | -0.13 | 8.39E-05 |
| cg08368934 | 16 | 57701455 | GPR97 | 6.48E-05 | 2.43E-01 | -- | -0.11 | -0.20 | -0.14 | 8.32E-05 |
| cg22381196 | 16 | 72041376 | DHODH | 3.61E-05 | 1.95E-01 | -- | -0.11 | -0.28 | -0.17 | 3.77E-05 |
| cg06627532 | 16 | 81491312 | CMIP | 3.99E-05 | 1.85E-01 | -- | -0.06 | -0.08 | -0.06 | 3.81E-05 |
| cg01704698 | 16 | 84941294 | CRISPLD2 | 4.52E-04 | 5.40E-02 | -- | -0.04 | -0.09 | -0.06 | 6.99E-05 |
| cg04656424 | 16 | 88839844 | FAM38A | 5.71E-05 | 2.42E-01 | -- | -0.07 | -0.09 | -0.07 | 7.46E-05 |
| cg13396713 | 17 | 505019 | VPS53 | 6.53E-05 | 2.38E-01 | -- | -0.12 | -0.16 | -0.14 | 8.10E-05 |
| cg04478251 | 17 | 998432 | ABR | 8.27E-05 | 1.87E-01 | -- | -0.10 | -0.13 | -0.11 | 6.99E-05 |
| cg13752093 | 17 | 1061129 | ABR | 4.70E-02 | 1.98E-05 | ++ | 0.02 | 0.01 | 0.02 | 4.41E-05 |
| cg12485428 | 17 | 1549098 | SCARF1 | 9.26E-05 | 2.19E-01 | -- | -0.10 | -0.13 | -0.11 | 9.54E-05 |
| cg19060895 | 17 | 5419436 | NLRP1 | 3.50E-05 | 2.38E-01 | -- | -0.08 | -0.14 | -0.10 | 4.91E-05 |
| cg07565956 | 17 | 7381288 | ZBTB4 | 6.74E-06 | 2.22E-01 | -- | -0.05 | -0.08 | -0.06 | 1.18E-05 |
| cg02025573 | 17 | 26972480 | KIAA0100 | 4.23E-03 | 1.98E-03 | -- | -0.01 | -0.01 | -0.01 | 3.77E-05 |
| cg26898932 | 17 | 28442480 | CCDC55 | 6.10E-05 | 1.27E-01 | -- | -0.11 | -0.21 | -0.14 | 3.26E-05 |
| cg26612727 | 17 | 38024636 | ZPBP2 | 4.88E-04 | 5.18E-02 | -- | -0.07 | -0.09 | -0.08 | 7.18E-05 |
| cg24312520 | 17 | 40489584 | STAT3 | 7.88E-04 | 2.27E-03 | ++ | 0.09 | 0.08 | 0.08 | 6.70E-06 |
| cg22396850 | 17 | 41121153 | AARSD1 | 1.57E-04 | 1.56E-01 | -- | -0.10 | -0.17 | -0.12 | 9.43E-05 |
| cg00620733 | 17 | 41723536 | MEOX1 | 1.78E-04 | 3.38E-02 | -- | -0.06 | -0.11 | -0.08 | 1.82E-05 |
| cg14648237 | 17 | 64422393 | PRKCA | 1.25E-04 | 1.82E-01 | -- | -0.09 | -0.18 | -0.12 | 9.54E-05 |
| cg04686354 | 17 | 73261880 | MRPS7 | 2.22E-05 | 2.19E-01 | -- | -0.09 | -0.20 | -0.13 | 3.00E-05 |
| cg23599820 | 17 | 73456199 | KIAA0195 | 6.59E-05 | 1.96E-01 | -- | -0.08 | -0.10 | -0.09 | 6.22E-05 |
| cg17346145 | 17 | 77755547 | CBX2 | 4.14E-05 | 2.43E-01 | -- | -0.09 | -0.15 | -0.11 | 5.80E-05 |
| cg14421700 | 17 | 77755965 | CBX2 | 7.87E-05 | 2.46E-01 | -- | -0.08 | -0.13 | -0.10 | 9.86E-05 |
| cg00483030 | 17 | 77810509 | CBX4 | 7.56E-05 | 2.46E-01 | -- | -0.12 | -0.18 | -0.14 | 9.57E-05 |
| cg07126783 | 17 | 78800767 | RPTOR | 4.14E-05 | 1.46E-01 | -- | -0.08 | -0.04 | -0.06 | 2.83E-05 |
| cg08885198 | 17 | 79051613 | BAIAP2 | 1.14E-02 | 1.97E-05 | ++ | 0.02 | 0.03 | 0.02 | 5.89E-06 |
| cg02802072 | 17 | 79229124 | SLC38A10 | 8.95E-05 | 2.19E-01 | -- | -0.09 | -0.15 | -0.11 | 9.29E-05 |
| cg09171638 | 17 | 80987627 | B3GNTL1 | 5.66E-03 | 9.80E-04 | -- | -0.02 | -0.04 | -0.03 | 3.15E-05 |
| cg19389293 | 18 | 13641872 | C18orf1 | 5.06E-06 | 2.47E-01 | -- | -0.12 | -0.13 | -0.12 | 1.11E-05 |
| cg16730908 | 18 | 43652592 | PSTPIP2 | 1.60E-04 | 1.45E-01 | -- | -0.05 | -0.07 | -0.06 | 8.73E-05 |
| cg08135234 | 18 | 60191519 | ZCCHC2 | 3.13E-03 | 2.47E-03 | ++ | 0.02 | 0.01 | 0.02 | 3.18E-05 |
| cg07564563 | 19 | 3548977 | C19orf28 | 1.81E-05 | 2.03E-01 | -- | -0.11 | -0.20 | -0.14 | 2.28E-05 |
| cg14402591 | 19 | 4543487 | SEMA6B | 1.48E-05 | 2.28E-01 | -- | -0.12 | -0.21 | -0.15 | 2.30E-05 |
| cg22218322 | 19 | 35085512 | SCGBL | 5.09E-04 | 1.25E-02 | -- | -0.03 | -0.05 | -0.04 | 1.86E-05 |
| cg17925829 | 19 | 36399328 | TYROBP | 3.21E-05 | 2.86E-01 | -- | -0.09 | -0.11 | -0.10 | 6.05E-05 |
| cg06432655 | 19 | 36523405 | CLIP3 | 3.30E-04 | 2.55E-02 | -- | -0.04 | -0.04 | -0.04 | 2.43E-05 |
| cg24376214 | 19 | 38918135 | RASGRP4 | 8.24E-04 | 2.00E-02 | ++ | 0.03 | 0.06 | 0.04 | 4.61E-05 |
| cg23990942 | 19 | 40946878 | SERTAD3 | 6.25E-05 | 2.40E-01 | -- | -0.08 | -0.16 | -0.11 | 7.91E-05 |
| cg14304515 | 20 | 822499 | FAM110A | 1.14E-05 | 2.73E-01 | -- | -0.05 | -0.10 | -0.07 | 2.47E-05 |
| cg12049875 | 20 | 19955868 | RIN2 | 6.72E-05 | 1.94E-01 | -- | -0.07 | -0.16 | -0.10 | 6.22E-05 |
| cg22821930 | 20 | 34330085 | RBM39 | 2.91E-03 | 4.43E-03 | ++ | 0.01 | 0.01 | 0.01 | 4.63E-05 |
| cg26688911 | 20 | 39969536 | LPIN3 | 1.81E-04 | 5.45E-02 | -- | -0.03 | -0.01 | -0.02 | 3.09E-05 |
| cg09868035 | 20 | 62492074 | C20orf135 | 1.91E-05 | 1.74E-01 | -- | -0.09 | -0.15 | -0.11 | 1.90E-05 |
| cg23085846 | 20 | 62522518 | TPD52L2 | 6.40E-06 | 2.78E-01 | -- | -0.09 | -0.17 | -0.11 | 1.62E-05 |
| cg10759927 | 20 | 62687969 | TCEA2 | 1.06E-04 | 1.43E-01 | -- | -0.06 | -0.08 | -0.07 | 6.02E-05 |
| cg15892280 | 21 | 40180000 | ETS2 | 3.75E-05 | 1.67E-01 | -- | -0.06 | -0.05 | -0.06 | 3.13E-05 |
| cg01542693 | 21 | 43528794 | UMODL1 | 3.23E-03 | 1.72E-03 | ++ | 0.06 | 0.07 | 0.06 | 2.51E-05 |
| cg22139500 | 21 | 45877719 | LRRC3 | 4.24E-07 | 3.73E-01 | ++ | 0.01 | 0.00 | 0.01 | 3.41E-06 |
| cg00236249 | 22 | 20789017 | SCARF2 | 3.33E-04 | 3.64E-02 | -- | -0.01 | -0.01 | -0.01 | 3.51E-05 |

Only CpGs with P<E-04 that map to a gene body are shown. Direction of beta was consistent in monozygotic (MZ) and dizygotic (DZ) twins. Chr: chromosome, RefGene: reference gene; Dir: direction; W: weighted.

| **Table S6. Most significant canonical pathways, upstream regulators, and diseases and biological functions in differentially methylated genes in whole blood from twin pairs discordant for dcSSc.** | |
| --- | --- |
| **Top canonical pathways** | **P-value** |
| Sonic Hedgehog Signaling | 3.20E-03 |
| **Top Upstream regulators** |  |
| WHSC1 | 3.01E-03 |
| **Top diseases and biological functions** |  |
| **Cancer, Gastrointestinal Disease, Organismal Injury and Abnormalities** | |
| intestinal tumor | 3.63E-05 |
| intestinal cancer | 4.87E-05 |
| large intestine neoplasm | 5.76E-05 |
| gastrointestinal tract cancer and tumors | 6.26E-05 |
| gastrointestinal tract cancer | 7.30E-05 |
| malignant neoplasm of large intestine | 7.79E-05 |
| **Cancer, Organismal Injury and Abnormalities** |  |
| adenocarcinoma | 1.51E-04 |
| **Cancer, Gastrointestinal Disease, Organismal Injury and Abnormalities** | |
| digestive organ tumor | 1.70E-04 |
| digestive system cancer | 1.96E-04 |
| **Cell Death and Survival** |  |
| apoptosis of T-cell hybrid cells | 3.12E-04 |
| **Cancer, Gastrointestinal Disease, Hepatic System Disease, Organismal Injury and Abnormalities** | |
| hepatocellular carcinoma | 4.04E-04 |
| **Cancer, Organismal Injury and Abnormalities** |  |
| epithelial cancer | 4.47E-04 |

| **Table S7. Reported SSc-associated gene regions with differentially methylated CpGs in SSc subsets.** | |
| --- | --- |
| **Associated gene region** | **Subset of differential methylation** |
| CD247 | All |
| HLA | All |
| IRF5 | dcSSc |
| RPL41-ESYT1 | All |
| NLRP1 | dcSSc |

| **Table S8. Differentially methylated CpG sites common to this study and to the report by Altorok et al (2015).** | | | | | | | | | | |
| --- | --- | --- | --- | --- | --- | --- | --- | --- | --- | --- |
| **CpG** | **Chr** | **Position (bp)** | **RefGene** | **Reported in this study** | | | | **Reported by Altorok *et al* (2015)** | | |
|  |  |  |  | Subset | Dir | W Beta | Meta P | Subset | Diff Score | Fold change |
| cg06580770 | 6 | 32054790 | TNXB | All | ++ | 0.03 | 7.52E-05 | dcSSc | -32.76 | 0.69 |
| cg00674995 | 13 | 42708444 | DGKH | All | ++ | 0.03 | 5.02E-05 | dcSSc | -63.16 | 0.59 |
| cg24062389 | 13 | 103478447 | BIVM | dcSSc | -- | -0.04 | 3.65E-05 | dcSSc | -22.37 | 0.46 |
| cg16558770 | 15 | 90548037 | ZNF710 | dcSSc | -- | -0.15 | 6.96E-05 | dcSSc | -22.64 | 0.69 |
| cg27018309 | 16 | 8943122 | PMM2 | lcSSc | -- | -0.02 | 6.70E-05 | dcSSc | -39.45 | 0.49 |
| cg07126783 | 17 | 78800767 | RPTOR | dcSSc | -- | -0.06 | 2.83E-05 | lcSSc | -25.41 | 0.69 |
| Chr: chromosome, RefGene: reference gene; Dir: direction; W: weighted; Diff: differential. | | | | | | | | | | |

| **Table S9. Cytosines differentially methylated in this study that are also reported as differentially methylated in blood from SLE patients.** | | | | | | | | | | | | | |
| --- | --- | --- | --- | --- | --- | --- | --- | --- | --- | --- | --- | --- | --- |
| CpG | Chr | Position | RefGene | This study | | | SLE | | | | | | |
|  |  |  |  | W Beta | Meta P-value | Disease subset | Naïve T cells | T cells | B cells | Monocytes | Neutrophils | WBCs | PBMCs |
| cg05599723 | 1 | 12241073 | TNFRSF1B | -0.13 | 8.03E-05 | dcSSc |  |  |  |  |  | hypo- | hyper- |
| cg23202722 | 1 | 33793808 | PHC2 | -0.15 | 6.67E-05 | dcSSc |  |  |  |  |  | hypo- | hyper- |
| cg16348470 | 1 | 34642609 | C1orf94 | -0.05 | 7.62E-05 | dcSSc |  |  |  |  |  |  | hyper- |
| cg07790169 | 1 | 49242932 | BEND5;AGBL4 | -0.01 | 3.38E-05 | All |  |  |  |  |  |  | hyper- |
| cg18854765 | 1 | 74665362 | LRRIQ3;TNNI3K;FPGT | -0.12 | 5.98E-05 | dcSSc |  |  |  |  |  |  | hyper- |
| cg03607951 | 1 | 79085586 | IFI44L | -0.12 | 8.85E-05 | All | hypo- | hypo- | hypo- | hypo- | hypo- |  | hyper- |
| cg03607951 | 1 | 79085586 | IFI44L | -0.17 | 5.60E-05 | dcSSc | hypo- | hypo- | hypo- | hypo- | hypo- |  | hyper- |
| cg03460527 | 1 | 95008117 | F3 | -0.01 | 4.19E-05 | lcSSc |  |  |  |  |  |  | hyper- |
| cg15346781 | 2 | 7017571 | RSAD2 | -0.06 | 9.29E-05 | All |  | hypo- | hypo- |  |  |  | hyper- |
| cg05057534 | 2 | 28497669 | BRE | -0.16 | 9.38E-05 | dcSSc |  |  |  |  |  |  | hyper- |
| cg00841141 | 2 | 37416819 | SULT6B1 | -0.15 | 7.27E-05 | dcSSc |  | hypo- |  |  |  |  |  |
| cg14480116 | 2 | 65594890 | SPRED2 | -0.16 | 2.84E-05 | dcSSc |  |  |  |  |  |  | hyper- |
| cg23527387 | 2 | 100056660 | REV1 | -0.05 | 7.91E-05 | dcSSc |  | hypo- |  |  |  |  |  |
| cg01462353 | 2 | 169939873 | DHRS9 | -0.14 | 7.95E-05 | dcSSc |  |  |  |  |  |  | hyper- |
| cg05364508 | 2 | 203242417 | BMPR2 | -0.02 | 9.84E-05 | All |  |  |  |  |  |  | hyper- |
| cg14414903 | 2 | 240171712 | HDAC4 | -0.11 | 8.12E-05 | dcSSc |  |  |  |  |  |  | hyper- |
| cg16967583 | 2 | 241807859 | AGXT | -0.11 | 9.89E-05 | dcSSc |  | hypo- |  |  |  |  |  |
| cg17619311 | 3 | 42947565 | ZNF662 | -0.06 | 1.77E-05 | All |  |  |  |  |  |  | hyper- |
| cg00218914 | 3 | 129146731 | C3orf25 | -0.09 | 7.16E-05 | dcSSc |  |  |  |  |  |  | hyper- |
| cg07258167 | 4 | 17514207 | QDPR | -0.04 | 7.37E-06 | dcSSc |  |  |  |  |  |  | hyper- |
| cg04747180 | 4 | 39116218 | KLHL5 | -0.14 | 9.68E-05 | dcSSc |  |  |  |  |  |  | hyper- |
| cg02394698 | 4 | 86594376 | ARHGAP24 | -0.14 | 7.14E-05 | dcSSc |  |  |  |  |  |  | hyper- |
| cg16700392 | 4 | 156680207 | GUCY1B3 | -0.04 | 6.65E-05 | lcSSc |  |  |  |  |  |  | hyper- |
| cg24866363 | 5 | 80050837 | MSH3 | -0.02 | 3.07E-06 | dcSSc |  |  |  |  |  |  | hyper- |
| cg19696103 | 5 | 132354130 | ZCCHC10 | -0.12 | 7.80E-05 | dcSSc |  |  |  |  |  |  | hyper- |
| cg24628744 | 5 | 134735654 | H2AFY | -0.09 | 2.89E-05 | dcSSc |  |  |  |  |  |  | hyper- |
| cg12699327 | 5 | 135170782 | LOC153328 | -0.01 | 1.39E-05 | All |  |  |  |  |  |  | hyper- |
| cg19972859 | 5 | 139494006 | PURA | -0.02 | 7.05E-05 | lcSSc |  |  |  |  |  |  | hypo- |
| cg10568066 | 6 | 30039442 | RNF39 | -0.11 | 9.27E-05 | dcSSc |  |  |  |  |  |  | hyper- |
| cg18924324 | 6 | 100057143 | PRDM13 | -0.03 | 6.49E-05 | dcSSc |  |  |  |  |  |  | hyper- |
| cg03120555 | 7 | 630473 | PRKAR1B | -0.13 | 3.85E-05 | dcSSc |  |  |  |  |  |  | hyper- |
| cg11884933 | 7 | 2774414 | GNA12 | -0.13 | 3.55E-05 | dcSSc |  |  |  |  |  |  | hyper- |
| cg14780416 | 7 | 27209338 | MIR196B | -0.01 | 9.76E-06 | All |  |  |  |  |  |  | hyper- |
| cg07302959 | 7 | 92198639 | FAM133B | -0.13 | 7.74E-05 | dcSSc |  |  |  |  |  |  | hyper- |
| cg05904013 | 7 | 128579933 | IRF5 | -0.11 | 9.67E-05 | dcSSc |  | hypo- |  |  |  |  | hyper- |
| cg12978800 | 7 | 151403164 | PRKAG2 | -0.06 | 9.73E-05 | dcSSc |  |  |  |  |  |  | hyper- |
| cg00219816 | 8 | 96280555 | C8orf37 | -0.07 | 2.16E-05 | dcSSc |  | hypo- |  |  |  |  |  |
| cg08598221 | 8 | 121824929 | SNTB1 | -0.09 | 2.10E-05 | dcSSc |  |  |  |  |  |  | hyper- |
| cg13630493 | 9 | 36190154 | CLTA | -0.08 | 3.03E-05 | dcSSc |  |  |  |  |  |  | hyper- |
| cg14364797 | 9 | 132651576 | FNBP1 | 0.04 | 1.84E-05 | All |  | hyper- |  |  |  |  | hypo- |
| cg26945715 | 10 | 96162066 | TBC1D12 | -0.03 | 9.13E-05 | All |  |  |  |  |  |  | hyper- |
| cg09767822 | 11 | 20178040 | DBX1 | -0.05 | 3.40E-05 | All |  |  |  |  |  |  | hyper- |
| cg20029347 | 11 | 46366877 | DGKZ | -0.04 | 6.83E-05 | All |  |  |  |  |  |  | hyper- |
| cg13912027 | 11 | 72759293 | FCHSD2 | -0.05 | 8.23E-05 | dcSSc |  |  |  |  |  |  | hyper- |
| cg09416908 | 11 | 86384670 | ME3 | -0.14 | 8.42E-05 | dcSSc |  |  |  |  |  |  | hyper- |
| cg20073686 | 11 | 105481863 | GRIA4 | -0.02 | 1.11E-05 | dcSSc |  |  |  |  |  |  | hyper- |
| cg01142676 | 11 | 117695591 | FXYD2 | -0.11 | 4.56E-05 | dcSSc |  |  |  |  |  |  | hyper- |
| cg26221631 | 11 | 129245988 | BARX2 | -0.02 | 7.65E-05 | All |  |  |  |  |  |  | hyper- |
| cg09802818 | 12 | 52604609 | LOC283404 | -0.11 | 2.25E-05 | dcSSc |  |  |  |  |  |  | hyper- |
| cg02566627 | 12 | 58232984 | CTDSP2 | -0.03 | 1.80E-05 | lcSSc |  |  |  |  |  |  | hyper- |
| cg07905054 | 12 | 102272136 | DRAM1 | -0.08 | 7.53E-05 | dcSSc |  |  |  |  |  |  | hyper- |
| cg04347477 | 12 | 125002007 | NCOR2 | -0.10 | 9.26E-06 | dcSSc |  |  |  |  |  |  | hyper- |
| cg17187521 | 12 | 125003379 | NCOR2 | -0.12 | 5.33E-05 | dcSSc |  |  |  |  |  |  | hyper- |
| cg26776551 | 13 | 51944507 | INTS6 | -0.13 | 4.78E-05 | dcSSc |  | hypo- |  |  |  |  | hyper- |
| cg22535089 | 13 | 114184462 | TMCO3 | -0.14 | 5.54E-05 | dcSSc |  |  |  |  |  |  | hyper- |
| cg05142272 | 14 | 24896538 | CBLN3 | -0.09 | 8.69E-05 | dcSSc |  |  |  |  |  |  | hyper- |
| cg17232357 | 15 | 67012832 | SMAD6 | -0.06 | 8.61E-05 | dcSSc |  | hypo- | hypo- | hypo- |  |  |  |
| cg03308839 | 16 | 15797297 | NDE1;MYH11 | -0.15 | 7.53E-05 | dcSSc |  |  |  |  |  |  | hyper- |
| cg08961793 | 16 | 28628118 | SULT1A1 | -0.13 | 8.39E-05 | dcSSc |  | hypo- |  |  |  |  | hyper- |
| cg22381196 | 16 | 72041376 | DHODH | -0.17 | 3.77E-05 | dcSSc |  |  |  |  |  |  | hyper- |
| cg12485428 | 17 | 1549098 | SCARF1 | -0.11 | 9.54E-05 | dcSSc |  |  |  |  |  |  | hyper- |
| cg07540103 | 17 | 6679499 | FBXO39 | -0.02 | 7.24E-05 | All |  |  |  |  |  |  | hyper- |
| cg26898932 | 17 | 28442480 | CCDC55 | -0.14 | 3.26E-05 | dcSSc |  |  |  |  |  |  | hyper- |
| cg12029804 | 17 | 38821390 | KRT222 | -0.04 | 6.94E-05 | All |  |  |  |  |  |  | hyper- |
| cg00620733 | 17 | 41723536 | MEOX1 | -0.08 | 1.82E-05 | dcSSc |  |  |  |  |  |  | hyper- |
| cg17346145 | 17 | 77755547 | CBX2 | -0.11 | 5.80E-05 | dcSSc |  |  |  |  |  |  | hyper- |
| cg14421700 | 17 | 77755965 | CBX2 | -0.10 | 9.86E-05 | dcSSc |  |  |  |  |  |  | hyper- |
| cg00483030 | 17 | 77810509 | CBX4 | -0.14 | 9.57E-05 | dcSSc |  |  |  |  |  |  | hyper- |
| cg07126783 | 17 | 78800767 | RPTOR | -0.06 | 2.83E-05 | dcSSc |  |  |  |  | hypo- |  |  |
| cg24682036 | 18 | 29077698 | DSG2 | -0.04 | 6.75E-05 | lcSSc |  |  |  |  |  |  | hyper- |
| cg12998614 | 19 | 917068 | KISS1R | -0.03 | 3.84E-06 | All |  |  |  |  |  |  | hyper- |
| cg09310112 | 19 | 4969989 | KDM4B | -0.01 | 7.36E-05 | All |  |  |  |  |  |  | hyper- |
| cg18592083 | 19 | 9731957 | ZNF561 | 0.00 | 6.64E-05 | lcSSc |  |  |  |  |  |  | hypo- |
| cg03202884 | 19 | 19248891 | TMEM161A | -0.04 | 7.60E-05 | lcSSc |  |  |  |  |  |  | hyper- |
| cg07756483 | 19 | 44281665 | KCNN4 | -0.02 | 4.99E-05 | All |  |  |  |  |  |  | hyper- |
| cg09868035 | 20 | 62492074 | C20orf135 | -0.11 | 1.90E-05 | dcSSc |  |  |  |  |  |  | hyper- |
| cg05602642 | 22 | 47081634 | CERK | -0.04 | 5.65E-05 | lcSSc |  |  |  |  |  |  | hyper- |

Chr: chromosome, RefGene: reference gene; W: weighted; WBC: white blood cells; PBMC: peripheral blood mononuclear cells.

| **Table S10. Most significant enrichment of top SSc CpGs overlapping cell-type-specific regulatory elements.** | | | | |
| --- | --- | --- | --- | --- |
| **Cell** | **Tissue** | **Datatype** | **Pvalue** | **Qvalue** |
| **DHS in cell and tissue types from ENCODE project data** | | | | |
| NS |  |  |  |  |
| **DHS in cell and tissue types from Roadmap Epigenomics project data** | | | | |
| Primary B cells from peripheral blood | Blood | H3K27me3 | 1.77E-06 | 0.00202 |
| **DHS in cell and tissue types from BLUEPRINT Epigenome data** | | | | |
| NS |  |  |  |  |
| **H3 marks in cell and tissue types from Roadmap Epigenomics data** | | | | |
| NS |  |  |  |  |
| DHS: DNase I hypersensitive sites; NS: none statistically significant. | |  |  |  |

| **Table S11. Most significant enrichment of top dcSSc CpGs overlapping cell-type-specific regulatory elements.** | | | | |
| --- | --- | --- | --- | --- |
| **Cell** | **Tissue** | **Datatype** | **Pvalue** | **Qvalue** |
| **DHS hotspots in cell and tissue types from ENCODE project data** | | | | |
| HL-60 | Blood | DHS | 7.22E-17 | 4.88E-14 |
| CD14+ | Blood | DHS | 4.71E-14 | 1.59E-11 |
| CD34+ | Blood | DHS | 5.93E-13 | 1.34E-10 |
| NB4 | Blood | DHS | 5.82E-11 | 9.83E-09 |
| HAEpiC | Epithelium | DHS | 3.34E-08 | 4.52E-06 |
| HCM | Heart | DHS | 3.43E-07 | 3.86E-05 |
| HSMM | Muscle | DHS | 7.82E-07 | 7.55E-05 |
| HPAF | Blood vessel | DHS | 1.16E-06 | 0.000087 |
| HVMF | Connective | DHS | 1.06E-06 | 0.000087 |
| PanIsletD | Pancreas | DHS | 3.51E-06 | 0.000237 |
| AG04450 | Lung | DHS | 6.28E-06 | 0.000386 |
| HCPEpiC | Epithelium | DHS | 6.89E-06 | 0.000388 |
| SKMC | Muscle | DHS | 0.0000103 | 0.000536 |
| NHEK | Skin | DHS | 0.0000123 | 0.000595 |
| HFF-Myc | Foreskin | DHS | 0.0000156 | 0.000704 |
| **DHS in cell and tissue types from Roadmap Epigenomics project data** | | | | |
| Primary monocytes from peripheral blood | Blood | DHS | 1.88E-09 | 3.12E-07 |
| Primary hematopoietic stem cells G-CSF-mobili | Blood | DHS | 0.0000157 | 0.000434 |
| Fetal Lung | Fetal Lung | DHS | 0.000011 | 0.000434 |
| Fetal Muscle Trunk | Fetal Muscle Trunk | DHS | 0.0000128 | 0.000434 |
| Placenta | Placenta | DHS | 0.0000147 | 0.000434 |
| Psoas Muscle | Psoas Muscle | DHS | 7.39E-06 | 0.000434 |
| H1 Derived Mesenchymal Stem Cells | ES Cell | DHS | 0.0000644 | 0.00153 |
| Foreskin Fibroblast Primary Cells | Skin | DHS | 0.000156 | 0.00324 |
| **DHS in cell and tissue types from BLUEPRINT Epigenome data** | | | | |
| inflammatory_macrophage | Venous Blood | DHS | 5.01E-20 | 6.00E-18 |
| macrophage | Venous Blood | DHS | 1.71E-14 | 1.02E-12 |
| inflammatory_macrophage | Venous Blood | DHS | 2.57E-13 | 1.03E-11 |
| Acute_myeloid_leukemia | Bone Marrow | DHS | 2.76E-12 | 5.52E-11 |
| macrophage_-_T_6days_B-glucan | Venous Blood | DHS | 2.00E-12 | 5.52E-11 |
| macrophage_-_T_6days_LPS | Venous Blood | DHS | 2.31E-12 | 5.52E-11 |
| CD14-positive_CD16-negative_classical_monocyte | Venous Blood | DHS | 3.23E-12 | 5.53E-11 |
| CD14-positive_CD16-negative_classical_monocyte | Venous Blood | DHS | 1.52E-11 | 2.28E-10 |
| macrophage_-_T_6days_B-glucan | Venous Blood | DHS | 2.58E-10 | 3.43E-09 |
| macrophage_-_T_6days_untreated | Venous Blood | DHS | 3.65E-09 | 4.37E-08 |
| CD14-positive_CD16-negative_classical_monocyte | Venous Blood | DHS | 5.35E-09 | 5.83E-08 |
| monocyte_-_T_0days | Venous Blood | DHS | 1.57E-08 | 1.57E-07 |
| CD14-positive_CD16-negative_classical_monocyte | Venous Blood | DHS | 2.62E-06 | 2.42E-05 |
| macrophage_-_T_6days_untreated | Venous Blood | DHS | 4.13E-06 | 3.53E-05 |
| **H3 marks in cell and tissue types from Roadmap Epigenomics data** | | | | |
| Primary monocytes from peripheral blood | Blood | H3K4me1 | 1.12E-20 | 1.28E-17 |
| Primary hematopoietic stem cells G-CSF-mobili | Blood | H3K4me1 | 3.55E-13 | 2.03E-10 |
| IMR90 fetal lung fibroblasts Cell Line | Lung | H3K4me1 | 5.93E-13 | 2.26E-10 |
| Primary hematopoietic stem cells G-CSF-mobili | Blood | H3K4me1 | 1.59E-12 | 4.53E-10 |
| Fetal Intestine Large | Fetal Intestine Large | H3K4me1 | 2.20E-11 | 5.01E-09 |
| Fetal Intestine Small | Feta Intestine Small | H3K4me1 | 5.24E-11 | 8.54E-09 |
| Psoas Muscle | Psoas Muscle | H3K4me1 | 5.08E-11 | 8.54E-09 |
| Fetal Adrenal Gland | Fetal Adrenal Gland | H3K4me1 | 3.08E-10 | 4.39E-08 |
| Primary Natural Killer cells from peripheral | Blood | H3K4me1 | 4.86E-10 | 6.16E-08 |
| Foreskin Fibroblast Primary Cells | Skin | H3K4me1 | 7.91E-10 | 9.02E-08 |
| Fetal Muscle Leg | Fetal Muscle Leg | H3K4me1 | 5.99E-09 | 6.22E-07 |
| Fetal Lung | Fetal Lung | H3K4me1 | 7.56E-09 | 7.19E-07 |
| Primary hematopoietic stem cells G-CSF-mobili | Blood | H3K4me3 | 1.13E-08 | 9.90E-07 |
| Fetal Thymus | Fetal Thymus | H3K4me1 | 2.67E-08 | 2.18E-06 |
| Foreskin Fibroblast Primary Cells skin02 | Skin | H3K4me1 | 5.70E-08 | 4.34E-06 |
| DHS: DNase I hypersensitive sites. |  |  |  |  |

**References**

1. Coit P, Jeffries M, Altorok N, Dozmorov MG, Koelsch KA, Wren JD, Merrill JT, McCune WJ, Sawalha AH. Genome-wide DNA methylation study suggests epigenetic accessibility and transcriptional poising of interferon-regulated genes in naive CD4+ T cells from lupus patients. J Autoimmun. 2013,43:78-84.

2. Absher DM, Li X, Waite LL, Gibson A, Roberts K, Edberg J, Chatham WW, Kimberly RP. Genome-wide DNA methylation analysis of systemic lupus erythematosus reveals persistent hypomethylation of interferon genes and compositional changes to CD4+ T-cell populations. PLoS Genet. 2013,9:e1003678.

3. Coit P, Yalavarthi S, Ognenovski M, Zhao W, Hasni S, Wren JD, Kaplan MJ, Sawalha AH. Epigenome profiling reveals significant DNA demethylation of interferon signature genes in lupus neutrophils. J Autoimmun. 2015,58:59-66.

4. Yeung KS, Chung BH, Choufani S, Mok MY, Wong WL, Mak CC, Yang W, Lee PP, Wong WH, Chen YA *et al*. Genome-Wide DNA Methylation Analysis of Chinese Patients with Systemic Lupus Erythematosus Identified Hypomethylation in Genes Related to the Type I Interferon Pathway. PLoS One. 2017,12:e0169553.

5. Zhu H, Mi W, Luo H, Chen T, Liu S, Raman I, Zuo X, Li QZ. Whole-genome transcription and DNA methylation analysis of peripheral blood mononuclear cells identified aberrant gene regulation pathways in systemic lupus erythematosus. Arthritis Res Ther. 2016,18:162.
